# Thiol-reactive compound depletion reveals electrophile-dependent and independent anti-inflammatory constituents in *Saussurea costus*

**DOI:** 10.3389/fphar.2026.1795322

**Published:** 2026-05-04

**Authors:** Janika Welzel, Sanjaykumar Boddul, Luke P. Robertson, Louisa Brieskorn, Iro Chaitoglou, Olga D. Chuquimia, Mingmei Shang, Mingshu Zhang, Liyan Mei, Wen Xu, Louise Berg, Helena Idborg, Fredrik Wermeling, Zehuai Wen, Marina Korotkova, Runyue Huang, Ulf Göransson, Per-Johan Jakobsson

**Affiliations:** 1 Division of Rheumatology, Department of Medicine, Solna, Karolinska Institutet, and Karolinska University Hospital, Solna, and Centre for Molecular Medicine, Karolinska University Hospital, Stockholm, Sweden; 2 Pharmacognosy, Department of Pharmaceutical Biosciences, Uppsala University, Uppsala, Sweden; 3 Section of Rheumatology and Immunology Research, The Second Affiliated Hospital of Guangzhou University of Chinese Medicine (Guangdong Provincial Hospital of Chinese Medicine), Guangzhou, China; 4 Center for Clinical Research of Guangdong Provincial Hospital of Chinese Medicine, Guangzhou University of Chinese Medicine, Guangzhou, China

**Keywords:** Chinese traditional medicine, immunoassays, inflammation, rheumatoid arthritis, *Saussurea costus*, sesquiterpene lactones

## Abstract

**Background:**

Rheumatoid arthritis (RA) is an autoimmune disease characterized by chronic inflammation and joint damage. Despite available treatments, many patients fail to achieve adequate disease control, prompting interest in complementary approaches. In traditional Chinese medicine, multi-herbal formulations such as Formulation C (Qushi-Juanbi Granules) are used clinically to treat RA. Among its component plants, *S. costus* (*S. costus*) has long been recognized for its anti-inflammatory properties that have largely been attributed to reactive *α*-methylene-*γ*-lactone-containing sesquiterpene lactones. However, comparatively little is known about the activities of non-reactive compounds.

**Objective:**

This study aimed to investigate the anti-inflammatory and anti-arthritic potential of *S. costus* and to determine the contribution of reactive sesquiterpene lactones to this effect.

**Materials and methods:**

Extracts from Formulation C plants were screened in cell-based assays targeting RA-relevant pathways (NF-κB, NFAT, STAT3/STAT5), prostanoid formation in a synovial fibroblast cell line, and cytokine production in primary B cells. The aqueous extract of *S. costus* and an electrophile-depleted version were prepared by elution through thiol-bonded silica and tested *in vitro* and in the KRN T cell transfer arthritis model *in vivo*. Seven compounds were isolated from *S. costus* and their activity characterized *in vitro*.

**Results:**

Screening identified the aqueous extract of *S. costus* with potent *in vitro* activities, mainly affecting the NF-κB and NFAT pathways, and the B cell assay. This activity was lost upon electrophilic-compound depletion, and the sesquiterpene lactones costunolide and dehydrocostus lactone were identified as major active constituents. *In vivo*, the aqueous extract modestly reduced paw swelling during early disease phase, whereas the electrophile-depleted extract showed minimal activity.

**Conclusion:**

This study applies a straightforward thiol-reactive compound depletion method to explore the bioactive constituents of *S. costus*, a strategy that can be broadly applied to other natural product extracts. Our findings indicate that sesquiterpene lactones are major contributors to the overall anti-inflammatory activity of *S. costus*, while other, non-thiol-reactive compounds may also play a role for *in vivo* activity. Overall, these results provide a more detailed characterization of the bioactive profile of *S. costus* and support its future mechanistic and preclinical investigation for RA research.

## 1 Introduction

Rheumatoid arthritis (RA) is a chronic autoimmune disease characterized by persistent joint inflammation (synovitis), cartilage destruction, and musculoskeletal symptoms such as arthralgia (Maeda et al., 2022). RA arises from a combination of genetic and environmental risk factors and affects up to 1% of Europeans and 0.5% of Asians (Smolen et al., 2018). It involves complex interactions between cells of both the innate and adaptive immune systems (Jang et al., 2022). Autoreactive T and B cells play a central role in initiating inflammation through autoantibody production and cytokine release (Yamada, 2022). Fibroblast-like synoviocytes further perpetuate chronic inflammation by producing pro-inflammatory mediators such as prostaglandins (PG) and interacting with macrophages and osteoclasts (Bartok and Firestein, 2010). Key inflammatory signalling pathways implicated in RA include nuclear factor kappa B (NF-κB) (Liao et al., 2025), nuclear factor of activated T cells (NFAT) (Park et al., 2020), signal transducers and activators of transcription 3 (STAT3), and STAT5 (Malemud, 2013), which promote pro-inflammatory mediator production, and immune cell activation and survival.

Despite the availability of various types of disease-modifying antirheumatic drugs (DMARDs), a substantial proportion of patients remain difficult-to-treat, with persistent disease activity despite conventional and multiple biologic or targeted therapies (Nagy et al., 2021). Combination therapies of two or more drugs are often used and can improve disease outcomes but increase treatment costs, risk of drug-drug interactions, and adverse events (Costello et al., 2019; Pflugbeil et al., 2020). Thus, there is an ongoing need for safe and effective alternative or adjunctive therapies.

Traditional Chinese medicine (TCM) offers a complementary approach for RA treatment, with multi-herbal formulations commonly used alongside conventional therapies in Chinese clinics (Zhang et al., 2020). While studies evaluating the efficacy of TCM formulations as monotherapy in RA are limited, meta-analyses of randomized controlled trials examining different RA formulations have demonstrated that combining TCM with conventional treatments, such as methotrexate (MTX), leads to improved efficacy and safety profiles compared to conventional therapies alone (Xing et al., 2020; Chen XM. et al., 2018; Kwon et al., 2023). Furthermore, studies have shown that such combination strategies can achieve outcomes comparable to standard drug combinations in RA patients (Gong et al., 2021; Wu et al., 2020).

In this study, we investigated a clinically used TCM formulation for RA treatment, referred to as Formulation C (Qushi-Juanbi Granules, QSJB), consisting of nine plant species in defined ratios (Supplementary Material, Supplementary Table S1). This formulation is derived from the ancient herbal formula Juan Bi Tang, which is traditionally used to treat RA-related symptoms (Li et al., 2021). A randomized, double-blind, placebo-controlled clinical trial in RA patients with low disease activity involving Formulation C (QSJB) has been completed (registration number ChiCTR2100053894), showing decreased disease activity with good tolerability (unpublished results).

In the present work, we further investigated this formulation using a previously described bioassay-guided screening strategy (Welzel et al., 2025; Jakobsson et al., 2022). In brief, organic and aqueous extracts and fractions thereof were prepared from each individual plant and evaluated for their anti-inflammatory properties. A panel of cell-based assays was used reflecting RA-relevant signalling pathways, as well as prostanoid formation in a synovial fibroblast cell line, and cytokine and IgG production in primary human B cells. Through this screening, the aqueous extract of *S. costus* (syn. *Dolomiaea costus, Aucklandia lappa*; here referred to as *S. costus*) was identified as one of the most potent and broadly active components, mainly inhibiting NF-κB and NFAT signalling and cytokine production in primary B cells.

*S. costus* is native to China and other parts of Asia, and its dried root extract (Mu Xiang in TCM) has traditionally been used to treat a range of conditions (Chen et al., 2025; Kumari et al., 2024), including gastrointestinal disorders (Guo et al., 2014), inflammation (Lim et al., 2020; Pang et al., 2025), cancer (Hasson et al., 2018), and infections (Idriss et al., 2023). Many of the observed activities have been attributed to the sesquiterpene lactones costunolide and dehydrocostus lactone (Liu et al., 2021), both of which are highly abundant in the plant and frequently used as quality control markers in TCM. A key feature underlying the activity of both is the presence of *α*-methylene-*γ*-butyrolactone functionalities, which readily act as Michael acceptors and engage in covalent interactions with cysteine residues in proteins (Romagnoli et al., 2005; Cateni et al., 2006). While this reactivity underlies their broad range of biological activities, it also confers promiscuity, as they can react with multiple proteins, potentially leading to undesired side effects or toxicity (Peng et al., 2017; Lin et al., 2015). Many other *α*,*β*-unsaturated carbonyl-containing sesquiterpene lactones are found in *S. costus*. These highly reactive functional groups have attracted considerable scientific attention, and numerous studies have focused on these compounds (Kumari et al., 2024; D et al., 2015; Liang et al., 2024). Conversely, non-*α*-methylene-*γ*-butyrolactone-containing compounds from *S. costus* have been comparatively less explored, and whether these have important roles for the biological effects of *S. costus* remains unknown.

The aim of this study was to investigate the anti-inflammatory and anti-arthritic effects of the aqueous extract of *S. costus*, following initial screening of plants from Formulation C for their anti-inflammatory properties. Additionally, by selectively removing electrophilic constituents using thiol-bonded silica columns, we sought to elucidate the contribution of the *α*-methylene-*γ*-butyrolactone-containing compounds to the overall anti-inflammatory activity of this plant.

## 2 Materials and methods

### 2.1 Plant material

All plant material was provided by the Guangdong Provincial Hospital of Chinese Medicine in China and produced by Kangmei Pharmaceutical Co. Ltd., Guangdong, China. Harvesting and processing followed the guidelines outlined in the Chinese Pharmacopeia (Chinese Pharmacopoeia Commission, 2020).

### 2.2 General extraction and isolation procedures

#### 2.2.1 Extraction and fractionation of plant material

Extraction and fractionation were performed according to a previously described protocol (Welzel et al., 2025). One single batch was prepared for each plant species, and used in all subsequent experiments. Initially, dried and pulverized plant material (100 g) was extracted by overnight maceration in dichloromethane/methanol (1:1). The solvent was removed *in vacuo* to give the “organic extract” (A). The residual plant biomass was then sequentially extracted overnight with water followed by 60% acetonitrile in water. These aqueous phases were pooled and freeze-dried to give the “aqueous extract” (I). All extracts were stored in the dark at 4 °C until further processing.

Organic extracts were fractionated on a diol-bonded silica column (PF-DIOL, 30 μm, 40 g; Interchim) with seven fractions collected using stepwise elution with hexane 100% (B), hexane-ethyl acetate (5:1) (C), hexane-ethyl acetate (1:1) (D), hexane-ethyl acetate (3:7) (E), ethyl acetate (F), ethyl acetate-methanol (7:3) (G), and methanol (H). Aqueous extracts were fractionated by reversed-phase chromatography on a C_18_ column (Sfär C_18_, 30 g, 100 Å, 30 μm; Biotage) with four fractions collected using stepwise elution with 5% (J), 20% (K), 60% (L), and 100% (M) acetonitrile in 0.1% trifluoroacetic acid (TFA). All fractions were dried and reconstituted to 2 mg/mL in 10% DMSO for bioactivity screening.

From 5 g of the organic extract (A) of *S. costus*, the following fractions were obtained: B (0.1642 g), C (1.4842 g), D (0.2020 g), E (0.1563 g), F (0.1154 g), G (1.1742 g), and H (0.9504 g). From 4 g of the aqueous extract (I) of *S. costus*, the following fractions were obtained: J (2.32 g), K (78 mg), L (94 mg), and M (113 mg).

Active fractions were identified by bioassay-guided screening and subjected to semi-preparative HPLC for subfractionation (Kinetex XB-C18, 5 μm, 100 Å, 250 × 10.0 mm). Identified active subfractions were then characterized by ^1^H NMR and LC–MS to obtain spectral fingerprints. If further purification was needed, these fingerprints guided the isolation of larger quantities.

#### 2.2.2 General isolation procedures

All natural product isolation and extract purity analyses were conducted using a Shimadzu LC-20 system equipped with two LC-20AD pumps, a DGU-20A degasser, a SIL-20AC autosampler, a CTO-20A column oven, a SPD-M20A PDA, controlled by a CBM-20A (Shimadzu, Kyoto, Japan).

##### 2.2.2.1 Isolation of 1–3

For preparative isolation, the organic extract of *S. costus* (1.5 g) was mixed with C_18_ silica gel (1.5 g) and dried. This extract impregnated dry powder was then packed into an empty HPLC precolumn (10 × 20 mm). The precolumn was connected in series with an HPLC column (Phenomenex Kinetex XB-C_18_, 150 × 21.2 mm, 5 μM, 100 Å) and purified using a gradient from 0%–100% CH_3_CN (0.1% TFA) over 35 min at a flow rate of 9 mL/min, with fractions collected at 60 s intervals. The fraction eluting at 25 min (40 mg) was further purified using semi-preparative HPLC (Phenomenex Kinetex XB-C_18_, 250 × 10 mm, 5 μM, 100 Å) at a flow rate of 4 mL/min using isocratic 71% CH_3_CN (0.1% FA) over 16 min. Compound **1** (6 mg) eluted at 8 min, **2** (13 mg) eluted at 9 min, and **3** (2 mg) eluted at 11 min.

##### 2.2.2.2 Isolation of 4–7

For preparative isolation, the organic extract of *S. costus* (5 g) was purified on diol-bonded silica as described above. The fraction eluting with EtOAc-MeOH (7:3) (800 mg) was mixed with C_18_ silica gel (800 mg) and dried. This extract impregnated dry powder was then packed into an empty HPLC precolumn (10 × 20 mm) and then connected in series with an HPLC column (Phenomenex Kinetex XB-C_18_, 150 × 21.2 mm, 5 μM, 100 Å). This was purified using a gradient from 30%–100% CH_3_CN (0.1% TFA) over 40 min at a flow rate of 9 mL/min, with fractions collected at 60 s intervals. The fraction eluting at 27 min contained **6** (18 mg) and the fraction eluting at 32 min contained **7** (8 mg). Fractions eluting at 12 min (10 mg) and 18 min (5 mg) were separately purified further. The first fraction (10 mg) was purified using semi-preparative HPLC (Phenomenex Kinetex XB-C_18_, 250 × 10 mm, 5 μM, 100 Å) at a flow rate of 4 mL/min using isocratic 30% CH_3_CN (0.1% FA) over 16 min, yielding pure **5** (5 mg). The second fraction (5 mg) was purified using the same conditions but with 32% CH_3_CN (0.1% FA), yielding pure **4** (2 mg).

##### 2.2.2.3 Compound identification

Compound identification was performed with full 2D NMR analysis (^1^H, 13C, COSY, HSQC, HMBC, and NOESY) acquired at 298 K on a Bruker 600 MHz (TCI CRPHe TR-1H and 19F/13C/15N 5 mm-EZ CryoProbe) spectrometer (Bruker, Billerica, MA, United States). All experiments were Bruker standard. Comparisons with literature values were later performed (Re et al., 2018; Wang, 2007; Zorrilla et al., 2024; Yuuya et al., 1999; Abegaz, 1991; Donadel et al., 2005).

#### 2.2.3 Preparation of electrophile-depleted *S. costus* extract for *in vitro* and *in vivo* studies

For each preparation, ∼3 g of the aqueous extract of *S. costus* was dissolved in water to a concentration of 50 mg/mL. The pH was measured (4.8), then adjusted to 8.0 with NaOH. Three equivalents (∼9 g) of thiol-bonded silica gel (ISOLUTE® Si-Thiol, 40–60 μm; Biotage) were added to the solution and the mixture stirred for ∼2 h at RT. The slurry was decanted into an empty solid-phase-extraction (SPE) column and eluted sequentially with 50 mL of distilled water, 50 mL of 50% acetonitrile, and finally with 50 mL of acetonitrile. The eluents were combined, freeze-dried, and weighed. Both the crude aqueous and the electrophile-depleted extract of *S. costus* were dissolved in DMSO (20 mg/mL) and analysed with HPLC-UV to confirm that the peaks associated with dehydrocostus lactone and costunolide had been depleted (Supplementary Figure S1). The depleted extract was then dissolved in distilled water to a concentration of 45 mg/mL and the pH adjusted back to 4.8 with HCl. For *in vitro* testing, the crude aqueous and depleted extracts were diluted to a stock concentration of 2 mg/mL in 10% DMSO.

##### 2.2.3.1 HPLC-UV analysis

For purity confirmation of the electrophile-depleted and non-depleted extracts, samples were prepared to a concentration of 20 mg/mL in DMSO and analysed using HPLC-UV using a gradient from 5%–95% CH_3_CN (0.1% FA) at a flow rate of 0.3 mL/min (Phenomenex Kinetex XB-C_18_, 100 × 3 mm, 2.6 µM, 100 Å).

##### 2.2.3.2 HPLC-MS analysis

All analyses were performed in positive electrospray ionization mode on a Waters Xevo G2-XS QTOF coupled to an Acquity Premier UPLC system. The capillary voltage was set to 3.0 kV, cone voltage to 40 V, source temperature to 150 °C, desolvation temperature to 600 °C, cone gas flow to 50 L/h, and desolvation gas flow to 1200 L/h. Chromatographic separation was done on a Phenomenex Kinetex XB-C18 column (100 × 3 mm, 2.6 µm, 100 Å) at 0.6 mL/min using a linear gradient of 5%–95% acetonitrile over 13 min.

### 2.3 Analysis of cell reporter bioassays using flow cytometry

Reporter cell lines were established in-house using retroviral or lentiviral transduction with different reporter plasmids as described previously (Welzel et al., 2025). Jurkat-NF-κB-GFP, Jurkat-NFAT-GFP, U937-STAT5-GFP, HEK-STAT3-GFP, and SW982-NF-κB-GFP cell lines were generated following previously described protocols (Boddul et al., 2021; Iyer et al., 2022; Wei et al., 2014). Cells were stimulated and populations with high green fluorescent protein (GFP) signal (stable reporter expression) were sorted on a Sony SH800 Cell Sorter (Sony Corporation) and used for subsequent bioassays.

All cell reporter lines were cultured according to the supplier’s recommended conditions (ATCC). Plant extracts and fractions were tested at a nominal concentration of 100 μg/mL in 0.5%–1% DMSO. The aqueous and depleted extracts of *S. costus* were tested at a range of concentrations (1–100 μg/mL) in Jurkat and SW982 (SW) cell reporter assays. Isolated compounds were tested at a range of concentrations (0.5–100 µM) in 0.5%–1% DMSO to obtain IC_50_ values. Cells were stimulated with pathway-specific agents as described previously (Welzel et al., 2025), with additional experimental details provided in Supplementary Material, Supplementary Table S2. HEK-STAT3 and SW-NF-κB cells were seeded the day before treatment to allow attachment, whereas Jurkat-NF-κB, Jurkat-NFAT, and U937-STAT5 cells were seeded and treated on the same day. Non-stimulated cells, stimulated cells treated with reference inhibitors, and stimulated cells in 0.5%–1% DMSO (for normalization) were included as controls. Cells were incubated for 16 h (Jurkat-NFAT) or 24 h (HEK-STAT3, SW-NF-κB, Jurkat-NF-κB, and U937-STAT5). Following incubation, cells were resuspended in autoMACS Running Buffer (Miltenyi Biotec) and stained with the fluorescent dye 7-AAD (BD Biosciences) for viability evaluation. Cells were then analysed by flow cytometry (FACSVerse, BD Biosciences; CytoFLEX, Beckman Coulter) for viability (7-AAD negative cells) and GFP signal, gating on viable single cells using FlowJo software.

### 2.4 Cytokine and IgG production in primary B cells

CD19^+^ B cells were isolated from peripheral blood mononuclear cells (PBMCs) of healthy donors using MACS MicroBeads with positive selection (Miltenyi Biotec) as previously described (Sundstrom et al., 2021). Cell viability and purity were assessed by flow cytometry (Cytek Aurora 4L, Cytek Biosciences) following staining with LIVE/DEAD Fixable Violet Dead Cell Stain (Invitrogen) and BV711-conjugated anti-human CD20 antibody (clone 2H7, BD Biosciences), with purity and cell viability around 95%. Plant extracts and fractions were tested at a nominal concentration of 40 μg/mL in 0.2% DMSO. Isolated compounds were tested at a range of concentrations (1–40 µM) in 0.2% DMSO. Cells were stimulated with activating agents as described previously (Welzel et al., 2025), with additional experimental details provided in Supplementary Material, Supplementary Table S2. Non-stimulated cells, stimulated cells treated with a reference inhibitor, and stimulated cells in 0.2% DMSO (for normalization) were included as controls. Cells were incubated for 22 h for cytokine production and 96 h for IgG production. Cytokine levels (interleukin (IL)-6, tumor necrosis factor (TNF)-α, and granulocyte-macrophage colony-stimulating factor (GM-CSF)) were determined in culture supernatants using the Luminex Performance Human High Sensitivity Cytokine Panel B (R&D Systems). IgG levels were measured using the ELISA Flex: Human IgG (ALP) kit (Mabtech). Cell viability was measured in parallel using the CellTiter-Glo Luminescent Cell Viability Assay (G7571, Promega).

### 2.5 Prostanoid profiling in synovial fibroblast cell line using LC-MS/MS

Prostanoid levels were determined from supernatants of stimulated SW-NF-κB cells treated with isolated compounds and IC_50_ values were calculated. Cells were stimulated with activating agents and incubated for 24 h as described previously (Welzel et al., 2025), with additional experimental details provided in Supplementary Material, Supplementary Table S2. Non-stimulated cells, stimulated cells treated with a reference inhibitor, and stimulated cells in 0.5% DMSO (for normalization) were included as controls. Supernatants were collected, spiked with deuterated prostanoid internal standards (PGE_2_-d4, PGD_2_-d4, TxB_2_-d4, and PGF_2α_-d4) (Cayman Chemical), and extracted by solid-phase-extraction (SPE) as previously described (Welzel et al., 2025; Bergqvist et al., 2019). Following acidification with 0.1% FA, samples were loaded onto Oasis HLB 96-well SPE plates (30 mg, 30 μm; Waters), washed with 5% methanol (0.05% FA), and eluted with 100% methanol. The eluates were dried, stored at −20 °C, and reconstituted in 20% acetonitrile prior to LC–MS/MS analysis (Acquity UPLC system coupled to a TQ detector, Waters). Chromatographic separation was done on an Acquity Premier BEH C_18_ column (1.7 µm, 130 Å, 2.1 × 50 mm) using a linear gradient from 20% to 80% acetonitrile (0.05% FA). Data acquisition and quantification were performed with MassLynx v4.2, using calibration with internal standards and external standard curves.

### 2.6 Mouse KRN T cell transfer arthritis model

Male and female B6.KRN and TCRβ^−/−^ I-A^b^/I-A^g7^ mice, aged 12–16 weeks, were used in all experiments. The B6.KRN strain was generously provided by Diane Mathis and Christophe Benoist (Kouskoff et al., 1996). The TCRβ^−/−^ knockout (KO) mice (stock no. 002118) and non-obese diabetic (NOD) mice (stock no. 001976) were obtained from Jackson Laboratory. The TCRβ^−/−^ I-A^b^/I-A^g7^ strain was generated in-house by crossing TCRβ^−/−^ KO mice with NOD mice. All procedures were conducted in accordance with ethical guidelines for the humane use of animals and were approved by the Regional Ethical Committee in Stockholm, Sweden (#7544-2024). The animals were housed under pathogen-free conditions in standard cages with a 12 h light-dark cycle and *ad libitum* access to standard chow food and water.

The KRN T cell transfer model of arthritis was employed as described previously (LaBranche et al., 2010). In brief, splenocytes were intravenously transferred into TCRβ^−/−^ I-A^b^/I-A^g7^ recipient mice to induce arthritis from days 3–4 post-injection. Mice were randomly assigned to different groups (Maeda et al., 2022): *S. costus* crude aqueous extract (Smolen et al., 2018), *S. costus* electrophile-depleted extract (Jang et al., 2022), water (vehicle) (Yamada, 2022), MTX (Bartok and Firestein, 2010), PBS (vehicle) (Liao et al., 2025), Formulation C, or (Park et al., 2020) Formulation C without *S. costus*. Extracts were administered by daily oral gavage (500 μL, ∼1 g/kg) starting on day 2 post-induction. MTX (A6770, Sigma-Aldrich) was administered intraperitoneally (2 mg/kg) every second day.

Body weight and joint swelling were assessed daily by researchers blinded to the treatment using a visual scoring system (scores 0-3 per paw, maximum score 12) as described previously (Monach et al., 2008). Mice were sacrificed at the peak of inflammation (day 9) or when they reached the maximum clinical score (humane endpoint). Serum samples were collected for analysis of anti-glucose-6-phosphate isomerase (anti-GPI) IgG and IL-6 levels.

### 2.7 ELISA

#### 2.7.1 Anti-GPI IgG

Serum anti-GPI IgG levels were measured by quantitative enzyme-linked immunosorbent assay (ELISA) as described previously (Welzel et al., 2025). In brief, 10 µg/well of the GPI protein (Merck) was coated on 96-well ELISA plates and incubated overnight at 4 °C. Plates were first blocked with blocking buffer, then samples added at 1:50 dilution and plates incubated for 2 h at RT on a rotating shaker. After incubation, the plates were first washed, then horseradish peroxidase-conjugated (HRP-conjugated) goat anti-mouse IgG (Santa Cruz Biotechnology) added and plates incubated for 2 h at RT on rotating shaker. After incubation, the plates were first washed, then developed by adding TMB substrate, and the reaction stopped by adding stop solution (Thermo-Fisher Scientific). Absorbance was measured at 450 nm on a plate reader.

#### 2.7.2 IL-6

Serum IL-6 levels were measured by quantitative ELISA (Mouse IL-6, ELISA MAX Standard Set, BioLegend) according to the manufacturer’s protocol. Samples were tested at 1:20 dilution.

### 2.8 Statistical analysis

All statistical analysis was performed using GraphPad Prism 9.0 software (CA, United States). IC_50_ values are shown as absolute IC_50_ values and were calculated based on GFP mean fluorescence intensities from n ≥ 2 independent experiments performed in duplicates and normalized to DMSO-treated activated control cells. If a GFP signal above 80% could not be achieved at the lowest tested concentration, a value of 100% was assigned at a 100-fold lower dilution, following GraphPad’s guidelines for determining absolute IC_50_ values. The specific statistical analyses used are detailed in the figure legends.

## 3 Results

### 3.1 Anti-inflammatory activities of formulation C plants in cell-based assays

**FIGURE 1:**
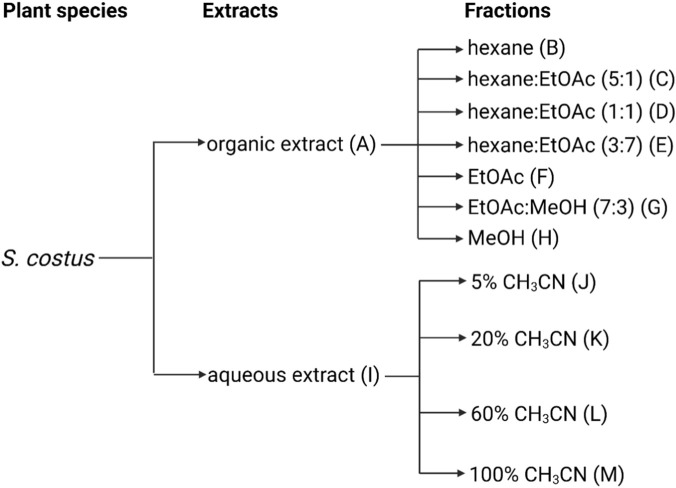
Overview of the fractionation of plant extracts. Extracts and fractions were prepared for each plant species from Formulation C, here exemplified for *S. costus*. EtOAc, ethyl acetate; MeOH, methanol; CH_3_CN, acetonitrile.

During the initial screening, we evaluated the inhibitory effects of the individual plants from Formulation C on different inflammatory pathways and cytokine production in primary B cells. Aqueous and organic extracts and their corresponding fractions were prepared for each plant species and tested at nominal concentrations of 100 μg/mL in reporter assays and 40 μg/mL in primary B cells (Figure 1). These concentrations were chosen as standardized screening conditions to facilitate identification of potential bioactive constituents while attempting to limit strong effects on cell viability. The extracts and fractions exhibited distinct inhibitory effects across the different assays and on cell viability (Supplementary Figures S2,S3). Screening data for *Angelica sinensis* and *Glycyrrhiza uralensis*, which are also components of another Chinese herbal formulation (Formulation A), have been reported previously (Welzel et al., 2025).

In general, the most active fractions were either derived from the organic extraction (fractions A-H) or from the aqueous extraction when eluted with 60% or 100% acetonitrile (fractions L and M) (Supplementary Figures S2, S3). These fractions frequently showed >50% inhibition on pathway activity (GFP signal <50% after normalization). However, they also often reduced cell viability (<50% after normalization), and the effects on the pathway could not be determined (Supplementary Figures S2, S3).

### 3.2 The aqueous extract of *S. costus* exhibits potent *in vitro* anti-inflammatory activity

**FIGURE 2:**
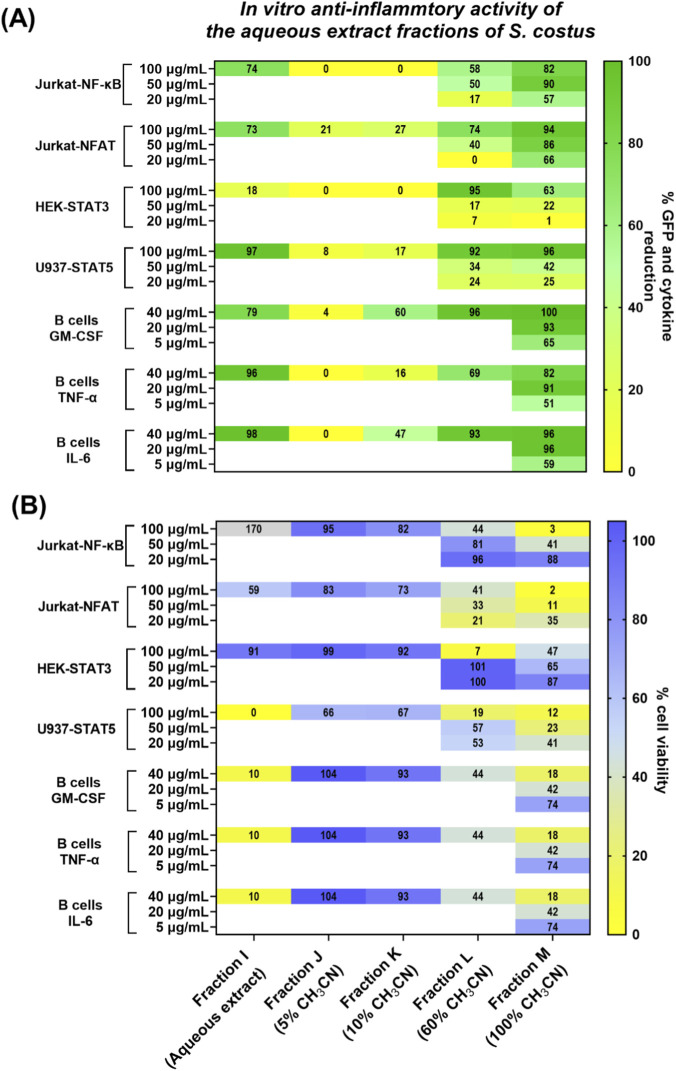
Anti-inflammatory activity of the aqueous extract fractions of *S. costus*. **(A)** The upper heatmap displays mean % GFP reduction in activated cell reporter assays (Jurkat-NF-ĸB, Jurkat-NFAT, HEK-STAT3, U937-STAT5) and mean % reduction in cytokine levels (GM-CSF, TNF-α, IL-6) in activated primary B cells. **(B)** The lower heatmap displays mean % cell viability. Values are shown within each cell. Data are normalized to stimulated control cells with DMSO (n = 1 experiment, n = 2 experiments for B cells at 40 μg/mL). Values > 100% reflect normalization to stimulated control and indicate increased viable cell number relative to control. White cells indicate conditions not tested.

Notably, the aqueous extract of *S. costus* (fraction I) showed anti-inflammatory activity in several cell-based assays, particularly inhibiting NF-κB and NFAT signaling in Jurkat cells (∼75% GFP reduction) while maintaining acceptable cell viability (>50%) (Figure 2, Supplementary Figure S2). In contrast, the majority of the organic fractions of *S. costus* (fractions A-F) strongly decreased cell viability across most assays (<50%), and pathway inhibition could not be ascertained at the tested concentration (Supplementary Figures S2, S3). Other aqueous extracts, including *L. chuangxiong* and *M. alba*, also showed inhibitory effects in specific assays, but these effects were generally accompanied by reduced cell viability (Supplementary Figures S2-S3). Based on its favourable activity and viability profile, the aqueous extract of *S. costus* and its fractions were selected for further investigation.

The effects of the aqueous extract of *S. costus* and its fractions on the STAT5 and B cell assays could not be reliably assessed at 100 μg/mL due to simultaneous strong reductions in cell viability (Figure 2, Supplementary Figure S2). However, inhibitory effects could be seen in the derived aqueous fractions L and M (Figure 2). Testing titrated concentrations revealed that these fractions largely retained potent pathway inhibition at lower concentrations in the Jurkat-NF-κB (20–50 μg/mL) and B cell cytokine assays (5 μg/mL), whereas higher concentrations resulted in decreased viability (Figure 2). These titration experiments should be considered as proof-of-concept studies (n ≥ 1) and were used to guide subsequent chemical analyses and studies of *in vivo* activity of the crude and electrophile-depleted extracts.

### 3.3 Anti-inflammatory activity of isolated compounds from *S. costus* in cell-based assays

**FIGURE 3:**
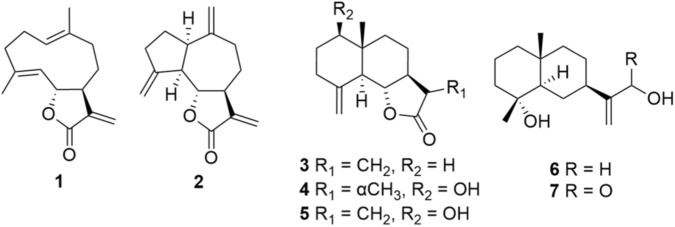
Isolated compounds from *S. costus*. Sesquiterpene lactones: Costunolide (1), dehydrocostus lactone (2), β-cyclocostunolide (3), dihydroreynosin (4), and reynosin (5). Sesquiterpenes: Ilicic alcohol (6) and ilicic acid (7).

After identifying the aqueous extract of *S. costus* with interesting bioactivities *in vitro*, we sought to investigate its molecular basis. Costunolide **(1)** and dehydrocostus lactone **(2)** were identified as the major constituents of the aqueous extract with HPLC-UV and LC-MS analysis (Supplementary Figures S1, S8–10). Although our biological investigations focused on the aqueous extract, additional compounds were isolated from the organic extract to provide a broader chemical overview of *S. costus*. Bioassay-guided fractionation followed by preparative and semi-preparative HPLC yielded seven purified compounds **(1–7)** (Figure 3). The isolated amounts ranged from 2 to 18 mg, with **1** and **2** obtained in the highest yields. Structural elucidation using 2D NMR and LC-MS confirmed identities consistent with reported literature (Re et al., 2018; Wang, 2007; Zorrilla et al., 2024; Yuuya et al., 1999; Abegaz, 1991; Donadel et al., 2005). All isolated compounds were subsequently evaluated in the aforementioned cell-based assays.

Among these, ilicic alcohol **(6)** and ilicic acid **(7)** showed no activity at the highest concentrations tested (50–100 µM) (Table 1). The remaining five compounds showed different potencies. Dihydroreynosin **(4)** was the least active, exhibiting effects only in the Jurkat-NFAT assay and on cytokine production at concentrations >50 µM (Table 1; Supplementary Figure S4). Reynosin **(5)** and β-cyclocostunolide **(3)** demonstrated moderate activity on the cell reporter assays, on prostanoid production in SW-NF-ĸB cells, and on cytokine and IgG production in primary B cells (Table 1; Supplementary Figure S4).

**TABLE 1:** IC_50_ values (in µM) of isolated compounds on pathway inhibition in activated cell reporter assays (n ≥ 3 experiments in duplicates) and on inhibition of prostanoid production (PGD_2_, PGE_2_, PGF_2α_, and TxB_2_) in activated SW-NF-ĸB cells (n ≥ 2 experiments in duplicates).

Compound assay	1	2	3	4	5	6	7	Inhibitor
SW-NF-ĸB	3.5^a^	1.8	28.4	n.e	22.2	n.e	n.e	4.3
Jurkat-NF-ĸB	1.5	1.5	12.1	n.d	8.2^a^	n.e	n.e	0.008
Jurkat-NFAT	n.d	2.2	(33.3)	(53.3)	15.4	n.e	n.e	0.08
HEK-STAT3	7.3	9.1^a^	45.1^a^	n.d	8.4	n.e	n.e	0.65
U937-STAT5	8.5^a^	(9.5)	45.5^a^	80.2^a^	39.1^a^	n.e	n.e	4.1
PGD_2_	0.4	(4.7)	14.6	n.e	13.6	n.e	n.e	0.005
PGE_2_	0.9	(4.7)	16.2	n.e	12.7	n.e	n.e	0.008
PGF_2α_	1.5	(4.2)	13.3	n.e	13.2	n.e	n.e	0.004
TxB_2_	1.9	(4.5)	11.5	n.e	13.5	n.e	n.e	0.007

The following reference inhibitors were used: Bay 11-7082 (SW-NF-ĸB); cyclosporin A (Jurkat-NF-ĸB, and Jurkat-NFAT); filgotinib (HEK-STAT3); STAT5 inhibitor ab141192 (U937-STAT5); and NS-398 (prostanoid production).

n.e., no effect up to 50 or 100 µM (<50% inhibition), n = 1 experiment.

n.d., not determined due to reduced cell viability at the tested concentration.

IC_50_ values in brackets indicate apparent values determined at concentrations where cell viability was ≤50%.

^a^ Cell viability was between 50%–75% at the reported IC_50_ value; for all other IC_50_ values the cell viability was >75%.

Dose-response curves corresponding to the IC_50_ determinations can be found in, Supplementary Figures S5–S7.

**FIGURE 4:**
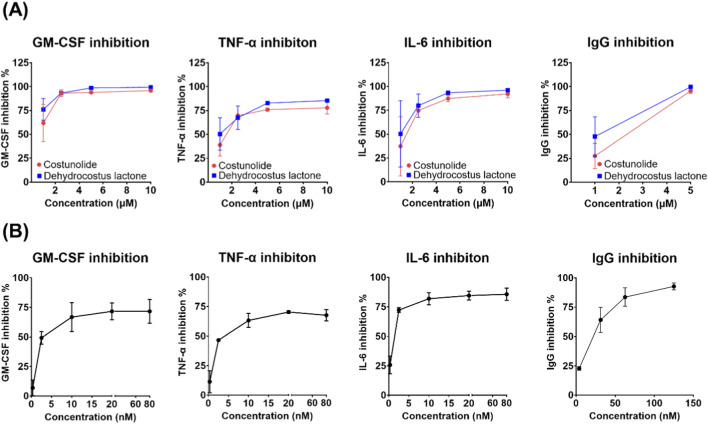
Costunolide and dehydrocostus lactone inhibit cytokine and IgG production in primary B cells. **(A)** Reduction in GM-CSF, TNF-α, IL-6 (n = 2 experiments), and IgG levels (n = 3 experiments) at different concentrations in activated primary B cells after treatment with costunolide (1) and dehydrocostus lactone (2). **(B)** Ibrutinib (for cytokine levels) and upadacitinib (for IgG levels) were used as reference inhibitors (n = 2 experiments). Data are presented as mean ± SEM. 1 and 2 simultaneously reduced cell viability (<50%) at concentrations ≥5 μM, and effects on cytokine and IgG production could not be reliably assessed.

**1** and **2** exhibited the broadest and most potent anti-inflammatory effects. Their IC_50_ values ranged from approximately 1.5–9.5 µM across cell reporter assays, with particularly strong inhibition in the Jurkat-NF-κB and SW-NF-κB assays (Table 1). Notably, **1** showed potent inhibition of all prostanoids (IC_50_ < 2 µM), whereas **2** reduced cell viability at the reported IC_50_ values (Table 1). Both compounds also affected cell viability in certain assays, particularly 2 in the U937-STAT5 and SW-NF-κB assays, and 1 in the Jurkat-NFAT assay. Both compounds also reduced IL-6, TNF-α, and IgG levels in primary B cells by about 50% at 1 μM, whereas concentrations ≥5 µM were generally associated with reductions in cell viability (<50%) (Figure 4).

### 3.4 The electrophile-depleted extract of *S. costus* shows no anti-inflammatory activity in cell-based assays

As described above, the sesquiterpene lactones **1** and **2** were identified as the major compounds of the aqueous extract of *S. costus* by HPLC-UV chromatography (Supplementary Figure S1). Although these compounds are commonly described as lipophilic, aqueous extracts are clinically relevant and widely used in traditional Chinese medicine. In this study, we aimed to investigate the chemical constituents relevant for bioactivity within the aqueous extract rather than to optimize extraction efficiency for individual compounds. To assess the activity of the extract after removal of these compounds, an electrophile-depleted version was prepared by eluting it through a thiol-bonded silica column. **1** and **2** were completely removed (>99%) and selectively depleted as judged by HPLC-UV (Supplementary Figure S1). Approximately 90% of the extract mass remained after elution, indicating that about 10% of the extract comprised compounds containing reactive *α*-methylene-*γ*-butyrolactone groups (Supplementary Figures S8–S10).

**FIGURE 5:**
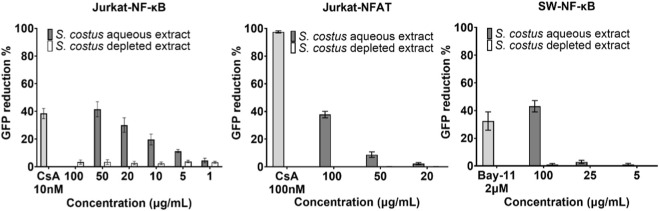
The electrophile-depleted extract shows no anti-inflammatory activity *in vitro*. Pathway inhibition was measured as reduction in GFP signal (mean ± SEM) in activated Jurkat-NF-ĸB, Jurkat-NFAT, and SW-NF-ĸB cells after treatment with different concentrations of the aqueous and the electrophile-depleted extract of *S. costus*. Data are normalized to stimulated control cells with DMSO (n = 3 experiments in duplicates). Cyclosporin A (CsA, Jurkat assays) and Bay-11 (SW-NF-ĸB assay) were used as reference inhibitors. Cell viability was ≥50% across all concentrations and assays, except at 100 μg/mL in the Jurkat-NF-ĸB assay, and pathway inhibition could not be reliably assessed.

The electrophile-depleted extract was then tested alongside the crude aqueous extract in the assays that previously showed the most pronounced inhibitory activity, namely, the Jurkat-NF-ĸB and Jurkat-NFAT assays. Both extracts were also tested in the SW-NF-ĸB assay to confirm effects in a second NF-κB reporter system. Notably, the crude aqueous extract of *S. costus* showed pronounced activity at 50–100 μg/mL in all assays, reducing the GFP signal to nearly 50% (Figure 5). In contrast, the electrophile-depleted extract did not show detectable activity at the tested concentrations (Figure 5).

### 3.5 The aqueous extract of *S. costus* modestly reduces joint inflammation in the KRN T cell transfer arthritis model

Although electrophile depletion resulted in a loss of *in vitro* activity, a potential functional role for non-thiol-reactive compounds could not be excluded, and prompted us to further investigate both extracts *in vivo* using the KRN T cell transfer arthritis model. This model is characterized by rapid disease onset and a severe polyarthritic phenotype that involves both priming and effector phases. The inflammation is mediated by T and B cell interactions, with subsequent pathogenic auto-antibody production against GPI (LaBranche et al., 2010; Christensen et al., 2016). In a pilot study, we tested Formulation C (which contains *S. costus*), the aqueous extract of *S. costus*, and Formulation C with *S. costus* removed in this model (Supplementary Figure S11). All treatments significantly reduced joint swelling compared to vehicle, with Formulation C and the aqueous extract of *S. costus* showing the most pronounced effects (Supplementary Figures S11C,D). In general, the extract alone demonstrated comparable anti-inflammatory efficacy to that of the whole formulation, suggesting an important role of *S. costus* in mediating the observed effects and guiding us to further explore its anti-arthritic effects *in vivo*.

**FIGURE 6:**
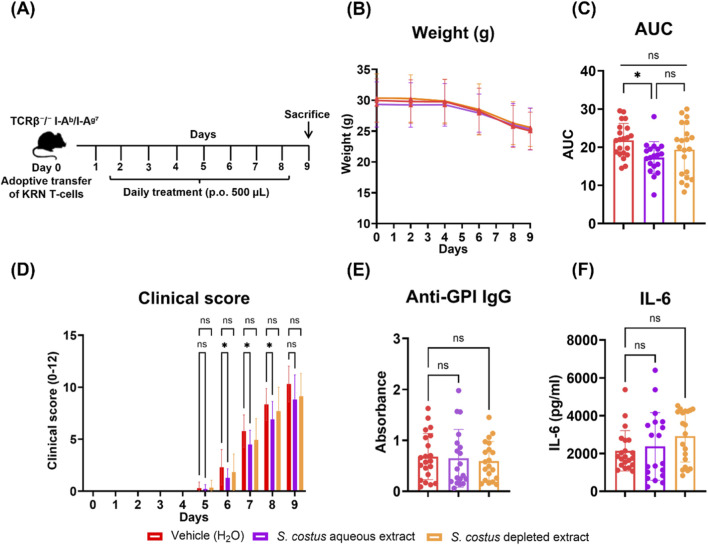
The aqueous extract of *S. costus* reduces joint swelling in the KRN T cell transfer arthritis model. Mice were randomly assigned to one of the following groups: Vehicle (n = 21), *S. costus* aqueous extract (n = 19), or electrophile-depleted *S. costus* extract (n = 21). **(A)** Induction of arthritis and treatment scheme. **(B)** Body weight. **(C,D)** Arthritis severity was assessed daily by an investigator blinded to the treatment. **(E,F)** At the endpoint, serum was analyzed for anti-GPI IgG antibody **(E)** and IL-6 levels **(F)** by ELISA. Data is presented as mean ± SD, pooling three independent experiments. *p < 0.05, **p < 0.01, and ns = not significant by two-way ANOVA **(D)** and one-way ANOVA **(C,E,F)**.

To assess the contribution of electrophilic sesquiterpene lactones to the observed *in vivo* effects, we tested the crude aqueous extract of *S. costus* alongside the electrophile-depleted one in this model (Figure 6), with MTX included as a positive control (Supplementary Figure S12). The crude aqueous extract significantly reduced arthritis scores on days 6–8 compared to vehicle (Figure 6D), corresponding to a reduction in overall disease severity of approximately 20% as measured by area under the curve (AUC) (Figure 6C). The effect was restricted to the early phase of disease progression and no significant differences were observed beyond day 8. The extract also did not alter serum anti-GPI IgG or IL-6 levels at the experimental endpoint (day 9) (Figures 6E,F). The electrophile-depleted extract showed limited and variable effects on joint swelling, with mean values of joint scores between those of the vehicle and crude extract groups. MTX treatment showed a significant suppression of joint swelling from days 6–9, with a mean reduction in AUC of approximately 65% (Supplementary Figures S12C,D). MTX also significantly reduced both serum anti-GPI IgG and IL-6 levels at the experimental endpoint (day 9) (Supplementary Figures S12E,F), which is consistent with its known mechanism of suppressing T and B cell activity and pro-inflammatory cytokine production (Pigott et al., 2014; Zhou et al., 2010). Body weight and general health remained stable across all groups, indicating good treatment tolerability (Figure 6B; Supplementary Figure S12B).

## 4 Discussion

### 4.1 Anti-inflammatory effects of *S. costus* and its constituents in cell-based assays

Previous studies have reported anti-inflammatory activity of the organic extract of *S. costus in vitro*, with several studies showing inhibitory effects on the production of pro-inflammatory mediators, including TNF-α, IL-1β, and IL-6, as well as cyclooxygenase-2 (COX-2), via suppression of NF-ĸB and mitogen-activated protein kinase (MAPK) signalling pathways (Lim et al., 2020; Jo et al., 2020). Consistent with these findings, we also observed marked anti-inflammatory activity of *S. costus* extracts and fractions. Interestingly, the aqueous extract displayed a particularly favourable profile, combining potent anti-inflammatory effects with retained cell viability in specific assays within a defined concentration range. Several fractions with strong effects on cell viability were also identified. While these fractions were not further investigated in this study, they may still be of interest in other research areas like cancer.

Among the identified bioactive compounds of *S. costus*, **1** is the most extensively studied, and previous reports have shown inhibition of NF-κB, STAT3, activator protein-1 (AP-1), and MAPK pathways *in vitro* (Butturini et al., 2011; Koo et al., 2001; Chen Z. et al., 2018). Similar biological activities have been demonstrated for **2** (Nie et al., 2019; Lee et al., 2020). Our findings are consistent with these reports, where **1** and **2** were identified as the most active compounds of *S. costus*. Comparable inhibitory effects in the Jurkat-NF-κB, Jurkat-NFAT, and SW-NF-κB assays were observed both for the isolated compounds and the crude extract, supporting their contribution in mediating these bioactivities. The reduction in cell viability observed for several of the *S. costus* fractions may also be in part mediated by **1** and **2**, which affected cell viability in several assays at the measured IC_50_ value. This overlap between pathway inhibition and reduced viability suggests a relatively narrow therapeutic window under the tested conditions and may limit translational potential unless selectivity can be improved. However, moderate reductions in viability do not exclude biological relevance, particularly in immune cell assays where pathway modulation and impaired survival may be mechanistically linked (Sarkar et al., 2008; Yu et al., 2009).

The activity of **1** and **2** is strongly mediated by the presence of *α*-methylene-*γ*-butyrolactone systems, characterized by an *α,β*-unsaturated carbonyl group with an exocyclic double-bond. This electrophilic group can covalently bind nucleophiles, e.g., cysteine thiols (-SH) in proteins via Michael addition (Liu et al., 2021). The minimal activity seen for **4** is presumably due to the reduction of its exocyclic double bond, eliminating the essential reactive electrophilic centre. Likewise, the lack of activity of **6** and **7** may be explained by the absence of the *α*-methylene-*γ*-butyrolactone ring system.

To our knowledge, this study is the first to apply such a broad panel of cell-based assays to assess the effects of *S. costus* on both signaling pathways and pro-inflammatory mediator production across multiple RA-relevant cell types.

### 4.2 Depletion of electrophilic thiol-reactive compounds from the aqueous extract of *S. costus*

Reactive sesquiterpene lactones like **1** and **2** often register activity in multiple assay types, a pattern consistent with broad, sometimes nonspecific, target engagement. Such promiscuous behaviour may categorize them as pan-assay interference compounds (PAINs) (Baell, 2016) and can complicate drug development due to increasing off-target effects and toxicity. Our *in vitro* results, comparing the activity of the crude aqueous extract of *S. costus* with that of the electrophile-depleted extract, support the promiscuous and highly reactive nature of these compounds. Removal of such compounds largely abolishes the *in vitro* activity, indicating that the observed effects of the extract are mainly attributable to these thiol-reactive constituents. Less-reactive compounds may still contribute to bioactivity, but their effects are not detected in the studied assays.

Although MS/UV-tagged thiol-based reagents have gained popularity in natural product research for the selective isolation of electrophilic compounds (Castro-Falcon et al., 2016), herein we describe a simple method to achieve the opposite. To our knowledge, we report the first application of thiol-functionalised silica columns for subtractive profiling of electrophilic natural products. Thiol-bonded silica is readily available commercially, primarily used for scavenging transition metals in synthetic chemistry (e.g., Pd, Cu, Ag). Depending on one’s perspective, Michael acceptors may be considered PAINs, often producing false-positive results in bioassays due to their promiscuous reactivity with cysteine residues (Baell, 2016). In such cases, a straightforward method like we introduce in this study for their selective removal could offer significant value in natural product research.

### 4.3 Evaluation of the aqueous and electrophile-depleted extracts of *S. costus* in the mice KRN T cell transfer arthritis model

Previous reports on the *in vivo* activity of *S. costus* have mainly focused on organic extracts, and have demonstrated anti-arthritic activity in different experimental models of arthritis (Jo et al., 2020; Tag et al., 2016). In the present study, we investigated the aqueous extract of *S. costus*, which showed a modest yet statistically significant reduction in joint swelling in the KRN arthritis model in the early phase of disease (days 6–8), but did not alter serum inflammatory markers (anti-GPI IgG and IL-6) at the experimental endpoint (day 9). The lack of significant effects on serum markers may relate to the timing of sampling at peak inflammation (day 9). In fact, the extract exerted its strongest effects earlier in the disease course, suggesting limited efficacy in later stages of inflammation.

Given the model’s aggressive disease course, detecting efficacy for moderately active treatments is challenging, and even MTX did not fully suppress inflammation. Differences in administration routes and dosing regimens may also influence efficacy, as MTX was given intraperitoneally at a relatively high clinical dose, whereas the extract was administered orally. Importantly, the aim of this study was to assess whether depletion of electrophilic constituents would alter the biological activity of the extract rather than to show therapeutic equivalence to standard antirheumatic drugs like MTX. Due to the small effect size and high variability within and between groups, the present *in vivo* findings should be considered exploratory and indicative of preliminary activity rather than definitive therapeutic efficacy. Considering the acute nature of this model, additional studies in chronic arthritis models, such as collagen-induced arthritis (CIA), may provide further insight into the extract’s activity under sustained inflammatory conditions and complement the current findings.

Notably, the electrophile-depleted extract showed only limited suppression of joint swelling in this model. While the pronounced *in vitro* activity of **1** and **2** suggests they are key mediators of the *in vivo* effects, the remaining activity after depletion indicates that non-thiol-reactive and less-characterized compounds may also play a role. However, given the mild efficacy of the crude aqueous extract, the contribution of these less-reactive compounds remains difficult to delineate. Future studies are warranted to isolate and characterize these compounds and to further explore their role for the overall activity of *S. costus*.

Although the aqueous extract demonstrated only modest efficacy *in vivo*, its favourable tolerability suggests a potential use as an adjunctive treatment. Combination strategies are widely used in RA management to improve efficacy and minimize adverse effects. For example, MTX in combination with leflunomide (LEF) has demonstrated effective disease control in RA patients, but hepatotoxicity remains a concern (Wang et al., 2018). Plant-derived compounds, such as total glucosides of paeony, have been reported to reduce the incidence and severity of liver injury associated with MTX/LEF therapy (Chen et al., 2013). Similarly, *Tripterygium wilfordii* Hook F in combination with MTX has shown improved remission rates compared to MTX alone (Lv et al., 2015). In this context, it would be of interest to explore whether *S. costus* could provide additive or synergistic benefit when combined with established antirheumatic treatments. Such approaches may allow dose reductions while maintaining therapeutic efficacy and improving safety.

## 5 Conclusion

In summary, the aqueous extract of *S. costus* exhibited strong anti-inflammatory activity *in vitro* and modest but measurable anti-arthritic effects in a highly aggressive and challenging murine arthritis model *in vivo*. This study advances previous work by systematically evaluating both crude and electrophile-depleted *S. costus* extracts. Our results identify the reactive sesquiterpene lactones costunolide and dehydrocostus lactone as major bioactive compounds *in vitro* and *in vivo*, while less-reactive and understudied compounds may also contribute to the observed *in vivo* effects.

Together, our findings reveal *S. costus* as a promising source of anti-inflammatory compounds and support its further preclinical evaluation, particularly in combination with existing therapies, in complementary arthritis models to better define its biological and therapeutic relevance for RA.

Finally, we introduce a straightforward subtractive approach using thiol-functionalised silica columns to selectively remove electrophilic constituents, offering a practical tool for dissecting bioactivity in complex natural product extracts and addressing the challenge of promiscuous Michael acceptors.

## Data Availability

The raw data supporting the conclusions of this article will be made available by the authors, without undue reservation.

## Glossary

AP-1: activator protein-1
AUC: area under curve
CH3CN: acetonitrile
CIA: collagen-induced arthritis
COX: cyclooxygenase
CsA: cyclosporin A
DMARDs: disease-modifying antirheumatic drugs
ELISA: enzyme-linked immunosorbent assay
EtOAc: ethyl acetate
FA: formic acid
FACS: fluorescence-activated cell sorting
GFP: green fluorescent protein
GM-CSF: granulocyte-macrophage colony-stimulating factor
GPI: glucose-6-phosphate isomerase
HCl: hydrochloric acid
HEK293T: HEK
HPLC: high-performance liquid chromatography
HRP: horseradish peroxidase
IgG: immunoglobulin G
IL: interleukin
KO: knockout
LC-MS: liquid chromatography-mass spectrometry
LEF: leflunomide
MACS: magnetic-activated cell sorting
MAPK: mitogen-activated protein kinase
MeOH: methanol
MS: mass spectrometry
MTX: methotrexate
NFAT: nuclear factor of activated T cells
NF-ĸB: nuclear factor kappa B
NMR: nuclear magnetic resonance
NOD: non-obese diabetic
PAINs: pan-assay interference compounds
PBMCs: peripheral blood mononuclear cells
PG: prostaglandin
Qushi-Juanbi Granules: QSJB
RA: rheumatoid arthritis
Saussurea costus: S. costus
SD: standard deviation
SEM: standard error of mean
SPE: solid-phase-extraction
STAT: signal transducers and activators of transcription
SW982: SW
TCM: traditional Chinese medicine
TFA: trifluoroacetic acid
TNF: tumor necrosis factor
UPLC: ultra-performance liquid chromatography.
