# Supplementary material for: Thiol-reactive compound depletion reveals electrophile-dependent and independent anti-inflammatory constituents in Saussurea costus
Source: Front Pharmacol. 2026 May 4;17:1795322. doi: 10.3389/fphar.2026.1795322 (PMC13180828; doi:10.3389/fphar.2026.1795322)
Supplement: Supplementary file 1 [file DataSheet1.pdf]

Supporting Information

Table S1: Composition of one dose of Formulation C (Qushi-Juanbi Granules).

Table S2: Treatment scheme for cell reporter lines.

Figure S1: HPLC-UV chromatogram of the aqueous extract of *S. costus*.

Figure S2: Effects of Formulation C plants on cell viability and GFP inhibition in different cell-based assays.

Figure S3: Effects of Formulation C plants on cell viability and cytokine production in primary human B cells.

Figure S4: Effects of β-cyclocostunolide (**3**), dihydroreynosin (**4**), and reynosin (**5**) on GM-CSF, TNF-α, IL-6, and IgG levels in primary human B cells.

Figure S5: Dose-response curves of reference control inhibitors on different cell-based assays.

Figure S6: Dose-response curves of bioactive compounds on different cell reporter assays.

Figure S7: Dose-response curves of bioactive compounds on prostaglandin production.

Figure S8: LC-MS comparison of the crude aqueous and electrophile-depleted extract of *S. costus*.

Figure S9: Zoomed LC-MS TIC for costunolide (**1**) in the crude aqueous and electrophile-depleted extract of *S. costus*.

Figure S10: Zoomed LC-MS TIC for dehydrocostus lactone (**2**) in the crude aqueous and electrophile-depleted extract of *S. costus*.

Figure S11: Pilot study on the effects of Formulation C, Formulation C without *S. costus*, and *S. costus* aqueous extract on joint inflammation in the KRN arthritis model.

Figure S12: Effects of MTX on joint inflammation in the KRN arthritis model.

Table S1: Composition of one dose of Formulation C (Qushi-Juanbi Granules).

Species	Family	Chinese name	Pinyin	Plant part	Mass (g)
Angelica sinensis (Oliv.) Diels	Apiaceae	当归	Dang gui	Root	10
Glycyrrhiza uralensis Fisch.	Fabaceae	炙甘草	Zhi gan cao	Root	15
Notopterygium incisum Ting ex H.T.Chang	Apiaceae	羌活	Qiang huo	Root	15
Gentiana macrophylla Pall.	Gentianaceae	秦艽	Qin jiao	Root	10
Kadsura heteroclita (Roxb.) Craib	Schisandraceae	海风藤	Hai feng teng	Root	30
Morus alba L.	Moraceae	桑枝	Sang zhi	Young stem	15
Ligusticum chuanxiong Hort.	Apiaceae	川穹	Chuan xiong	Root	15
Saussurea costus (Falc.) Lipsch.	Asteraceae	木香	Mu xiang	Root	10
Aconitum carmichaelii Debx.	Ranunculaceae	熟附子	Shu fu zi	Root	10

Table S2: Treatment scheme for cell reporter lines.

Cell assays	Stimulant(s)	Stimulant conc	Reference inhibitor	Inhibitor IC_{50}	Incubation time
**SW-NF-κB**	rh TNF-α (R&D Systems)	10 ng/mL	Bay 11-7082 (Sigma-Aldrich)	4.3 µM	24 h
	rh IL-1β (R&D Systems)	10 ng/mL	COX-2 inhibitor NS-398 (Merck)	0.1 µM	
**Jurkat-NF-κB**	anti-human CD3 (OKT-3 clone, Bio X cell)	1.25 µg/mL	cyclosporin A (Sigma-Aldrich)	7.9 nM	24 h
	anti-human CD28 (9.3 clone, Bio X cell)	1.25 µg/mL			
**Jurkat-NFAT**	ionomycin (Sigma-Aldrich)	0.5 µM	cyclosporin A	80.4 nM	16 h
	PMA (Sigma-Aldrich)	16 nM			
**HEK-STAT3**	rh LIF (PeproTech Inc.)	0.5 ng/mL	filgotinib (Cayman Chemical)	0.65 µM	24 h
**U937-STAT5**	rh GM-CSF (PeproTech Inc.)	0.4 ng/mL	STAT5 inhibitor (ab141192, Abcam)	4.1 µM	24 h
**B cells - Cytokines**	anti-human IgG and IgM H+L (Jackson ImmunoResearch)	10 µg/mL	pan-Bruton's kinase inhibitor ibrutinib (R&D Systems)	10 nM	22 h
	rh CD40 Ligand (R&D Systems)	1 µg/mL			
	rh IL-4 (R&D Systems)	2 ng/mL			
**B cells - IgG**	R848 (Resiquimod, InvivoGen)	1 µg/mL	JAK inhibitor upadacitinib (Cayman Chemical)	0.5-1 µM	96 h
	rh IL-2 (PeproTech Inc.)	10 ng/mL			
	rh IL-21 (PeproTech Inc.)	50 ng/mL			

Conc., concentration; rh, recombinant human; JAK, Janus Kinase

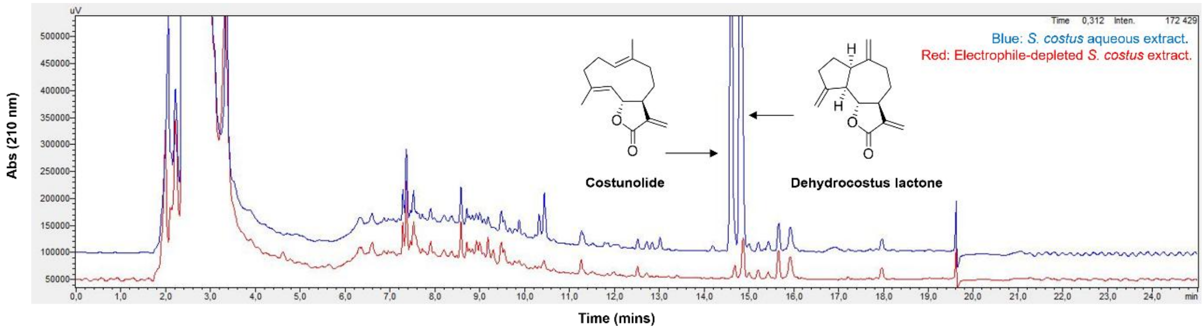

Figure S1. Overlaid HPLC-UV chromatograms of *S. costus* aqueous extract (blue line) compared with the electrophile-depleted *S. costus* aqueous extract after treatment with ISOLUTE® Si-Thiol columns. The peaks associated with the major sesquiterpene lactones, costunolide (**1**) and dehydrocostus lactone (**2**), are marked.

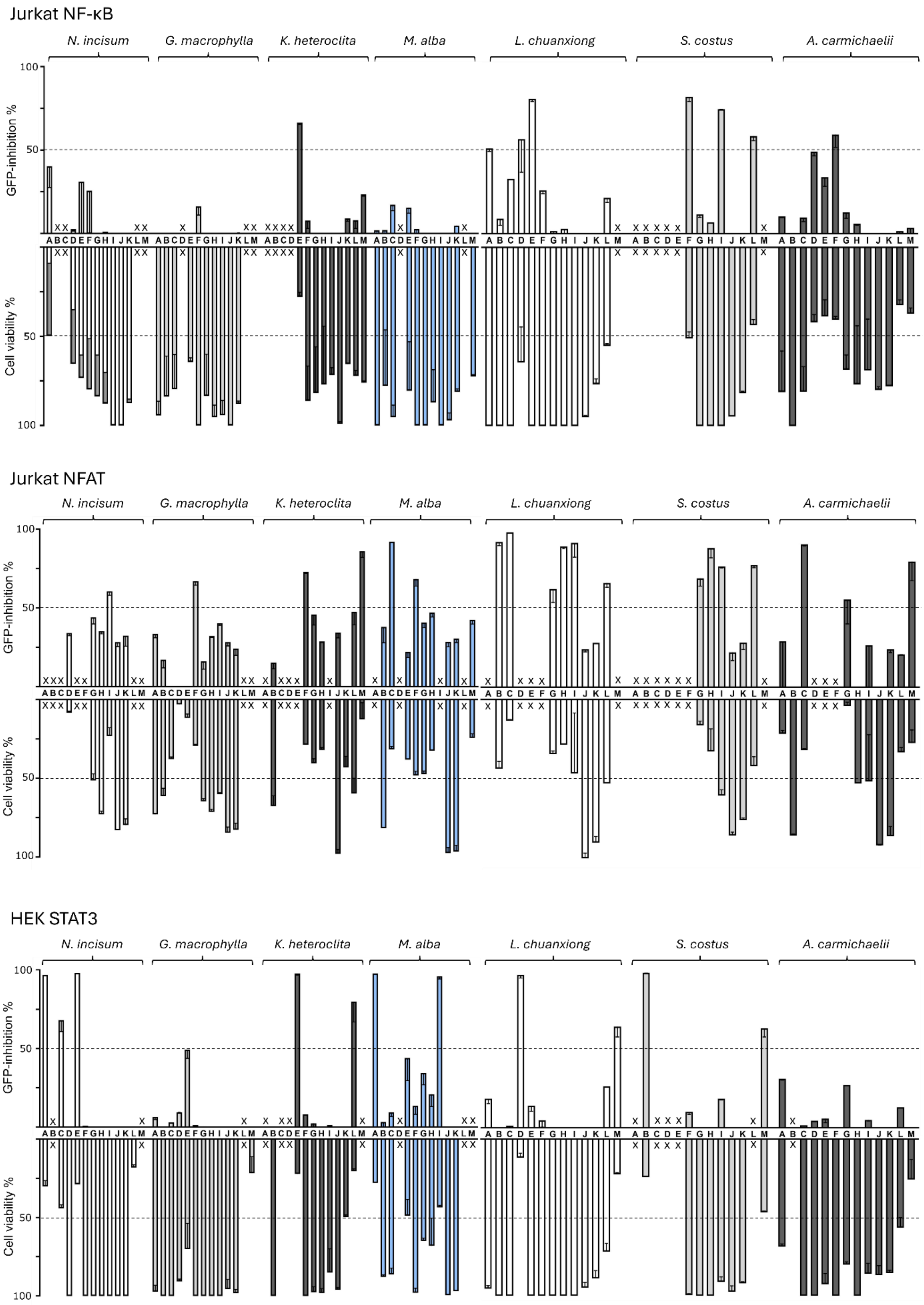
Jurkat NF-κB
N. incisum
G. macrophylla
K. heteroclita
M. alba
L. chuanxiong
S. costus
A. carmichaelii
GFP-inhibition %
Cell viability %
100
50
ABCDEFGHIJKLM
Jurkat NFAT
HEK STAT3

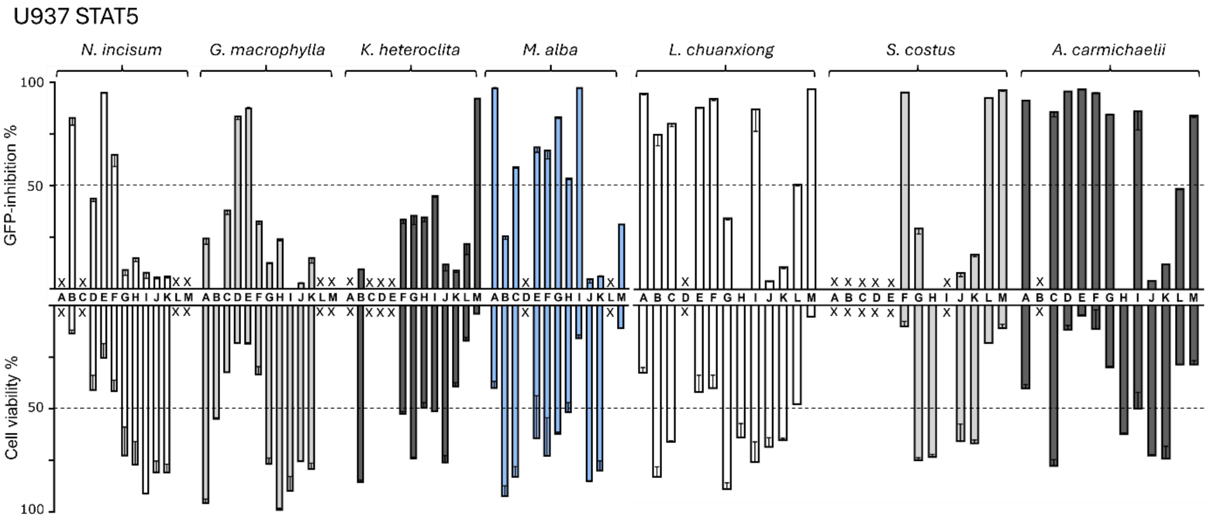

Figure S2: Effects of organic and aqueous extract fractions from Formulation C plants on cell viability and GFP inhibition in activated cell reporter assays (Jurkat-NF-κB, Jurkat-NFAT, HEK-STAT3, and U937-STAT5) at 100 µg/mL. A, organic extract; B, hexane; C, hexane:EtOAc (5:1); D, hexane:EtOAc (1:1); E, hexane:EtOAc (3:7); F, EtOAc; G, EtOAc:MeOH (7:3); H, MeOH; I, aqueous extract; J, 5% CH_3CN; K, 20% CH_3CN; L, 60% CH_3CN; M, 100% CH_3CN. Fractions marked with an x symbolize low number of viable cells (<1000 events) and thus the pathway activity (GFP signal) could not be ascertained. Data are presented as the mean ± SD (n=1 experiment in duplicates) normalized to stimulated control cells with DMSO.

IL-6

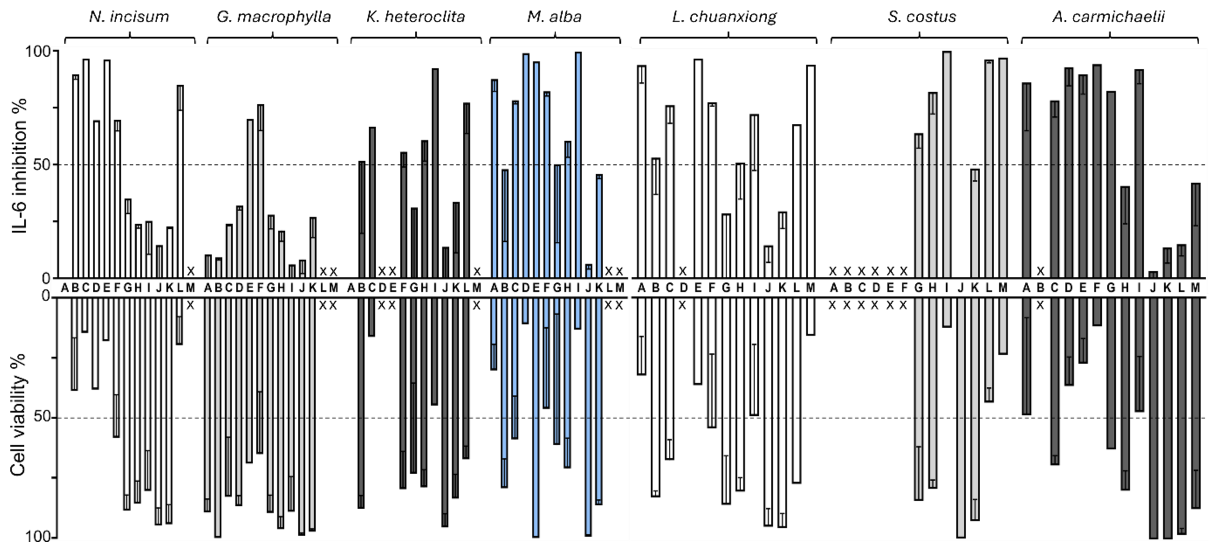

TNF-α

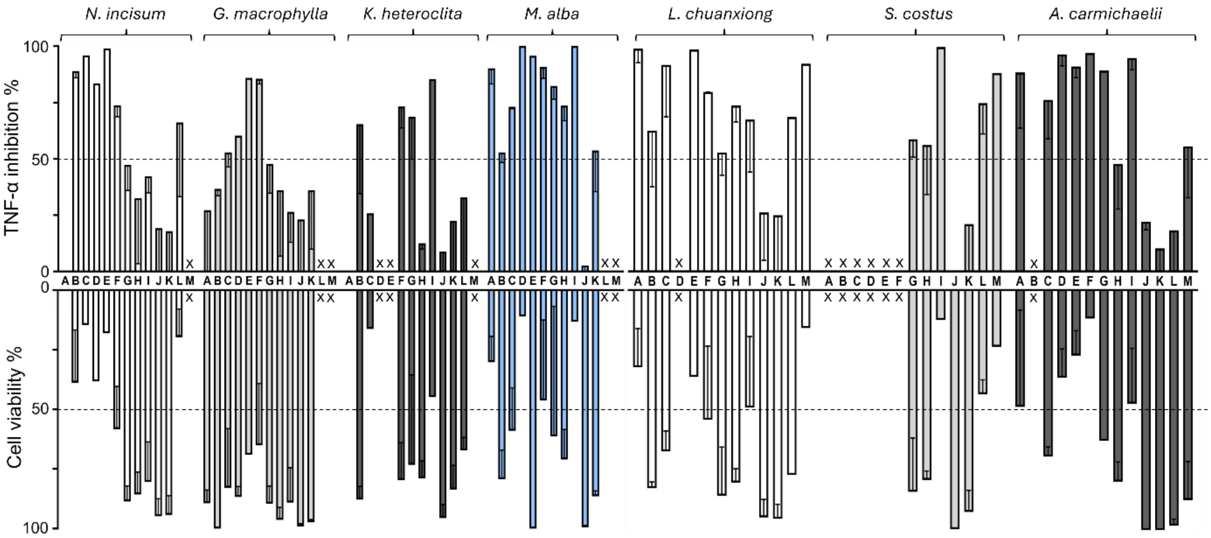

GM-CSF

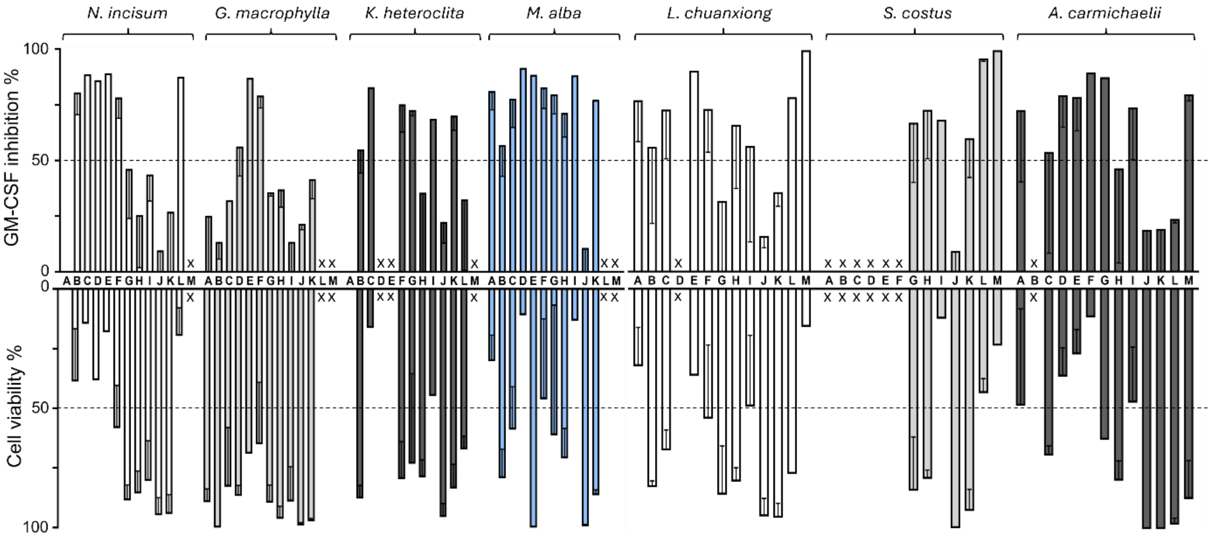

Figure S3: Effects of organic and aqueous extract fractions from Formulation C plants on cell viability, IL-6, TNF-α, and GM-CSF inhibition in activated primary B cells at 40 µg/mL. A, organic extract; B, hexane; C, hexane:EtOAc (5:1); D, hexane:EtOAc (1:1); E, hexane:EtOAc (3:7); F, EtOAc; G, EtOAc:MeOH (7:3); H, MeOH; I, aqueous extract; J, 5% CH_3CN; K, 20% CH_3CN; L, 60% CH_3CN; M, 100% CH_3CN. Fractions marked with an x symbolize strong reduction on cell viability (<10%) and thus cytokine levels could not be ascertained. Data are presented as the mean ± SD (n=2 experiments in duplicates) normalized to stimulated control cells with DMSO.

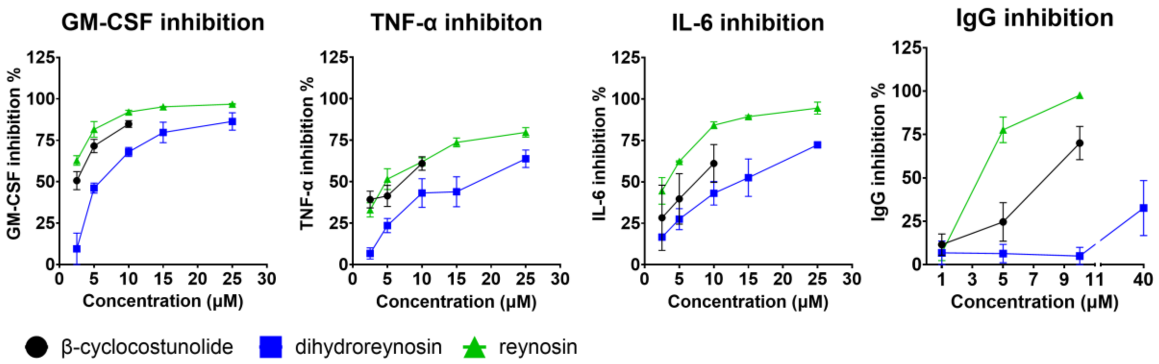

Figure S4: Reduction in GM-CSF, TNF-α, IL-6, and IgG levels (mean ± SEM) at different concentrations in activated primary B cells (n≥2 experiments in duplicates) after treatment with β-cyclocostunolide (**3**), dihydroreynosin (**4**), and reynosin (**5**). **5** simultaneously reduced cell viability (<50%) at 25 μM in cytokine assay and at 10 μM in IgG assay.

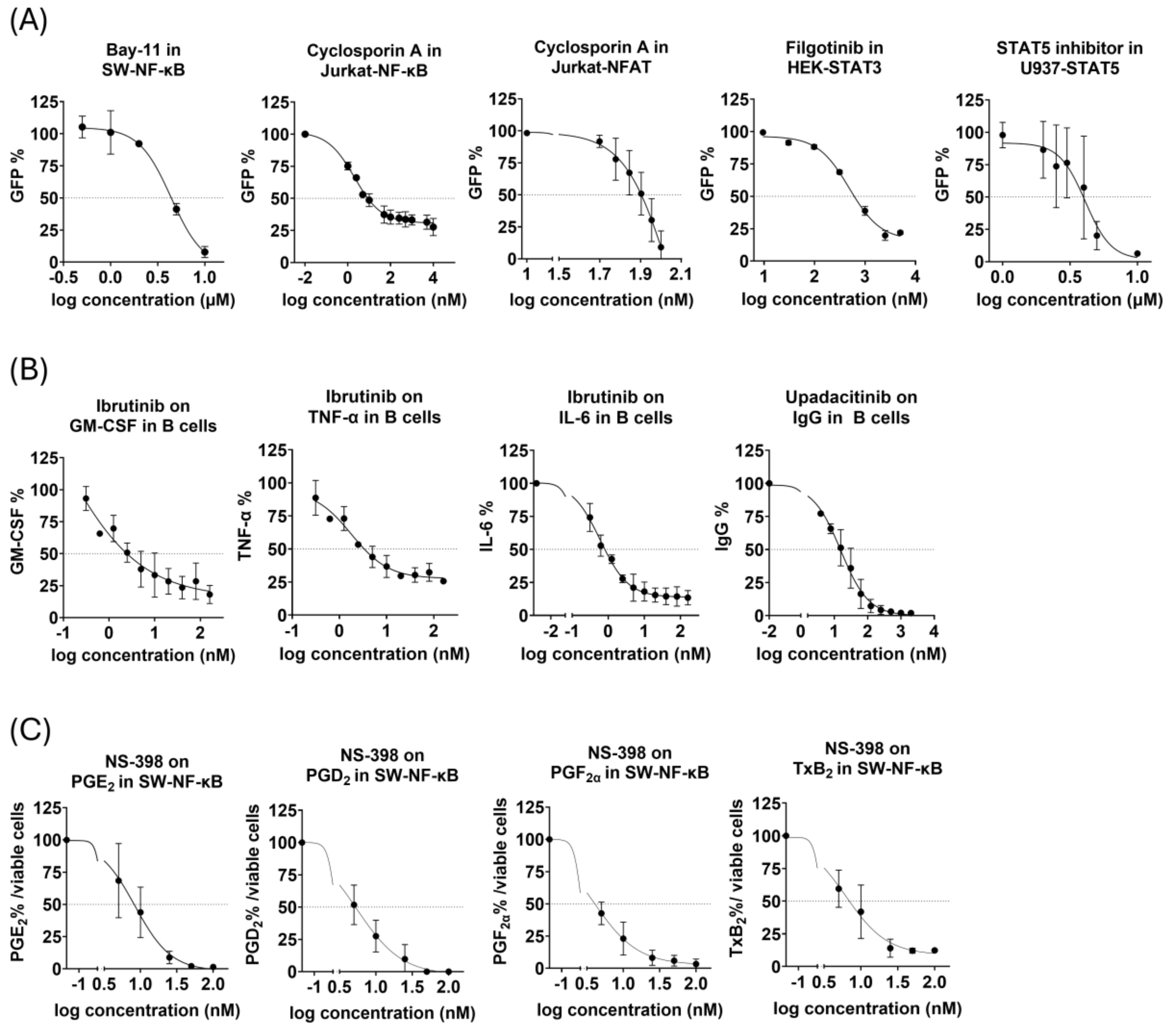

Figure S5: Dose-response curves of reference inhibitors in different cell-based assays: Cell reporter assays (A); cytokine and IgG levels in primary B cells (B); and prostaglandin levels in SW982-NF-κB cells (C).

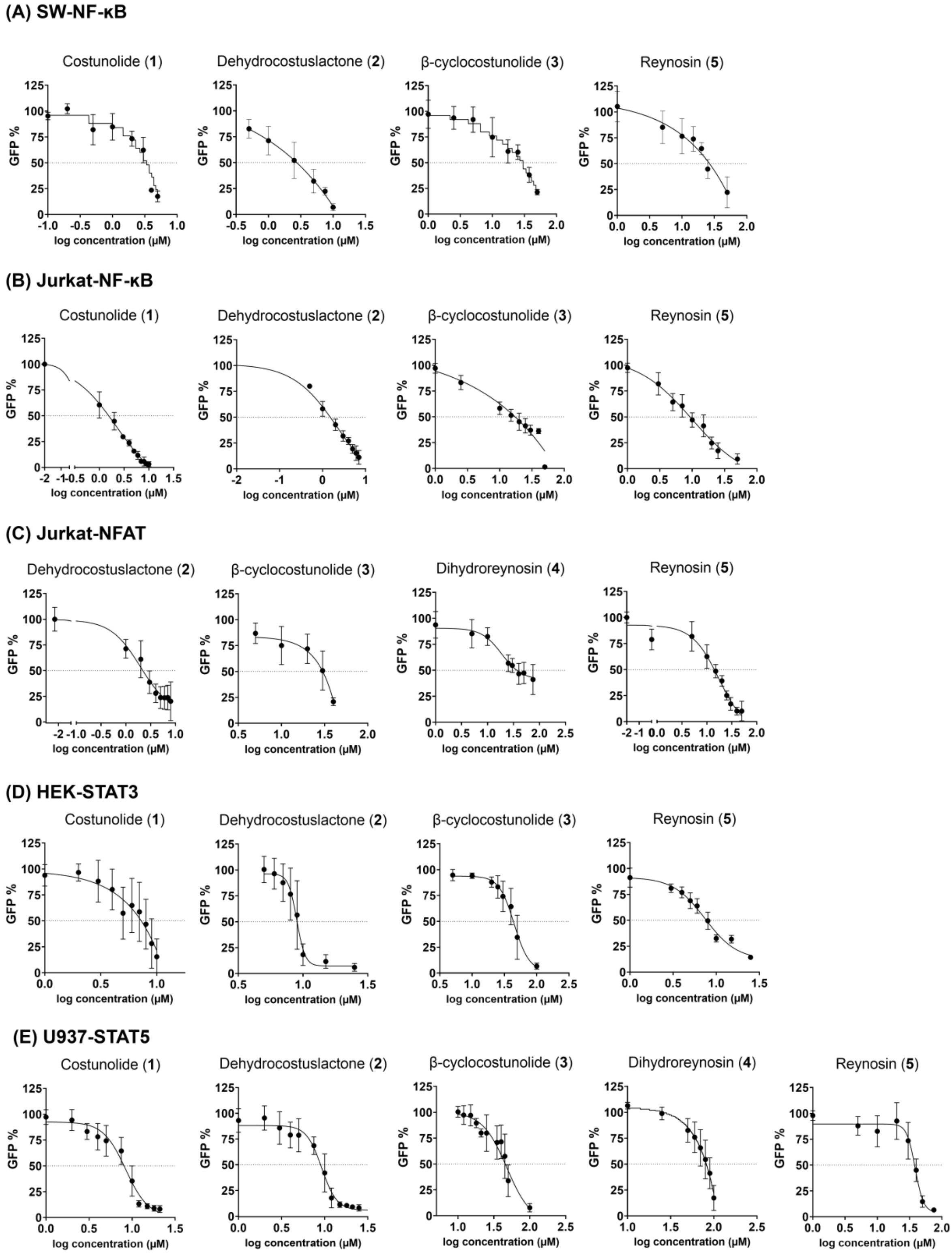

Figure S6: Dose-response curves of isolated active compounds on different cell reporter assays: SW-NF-κB (A), Jurkat-NF-κB (B), Jurkat-NFAT (C), HEK-STAT3 (D), and U937-STAT5 (E).

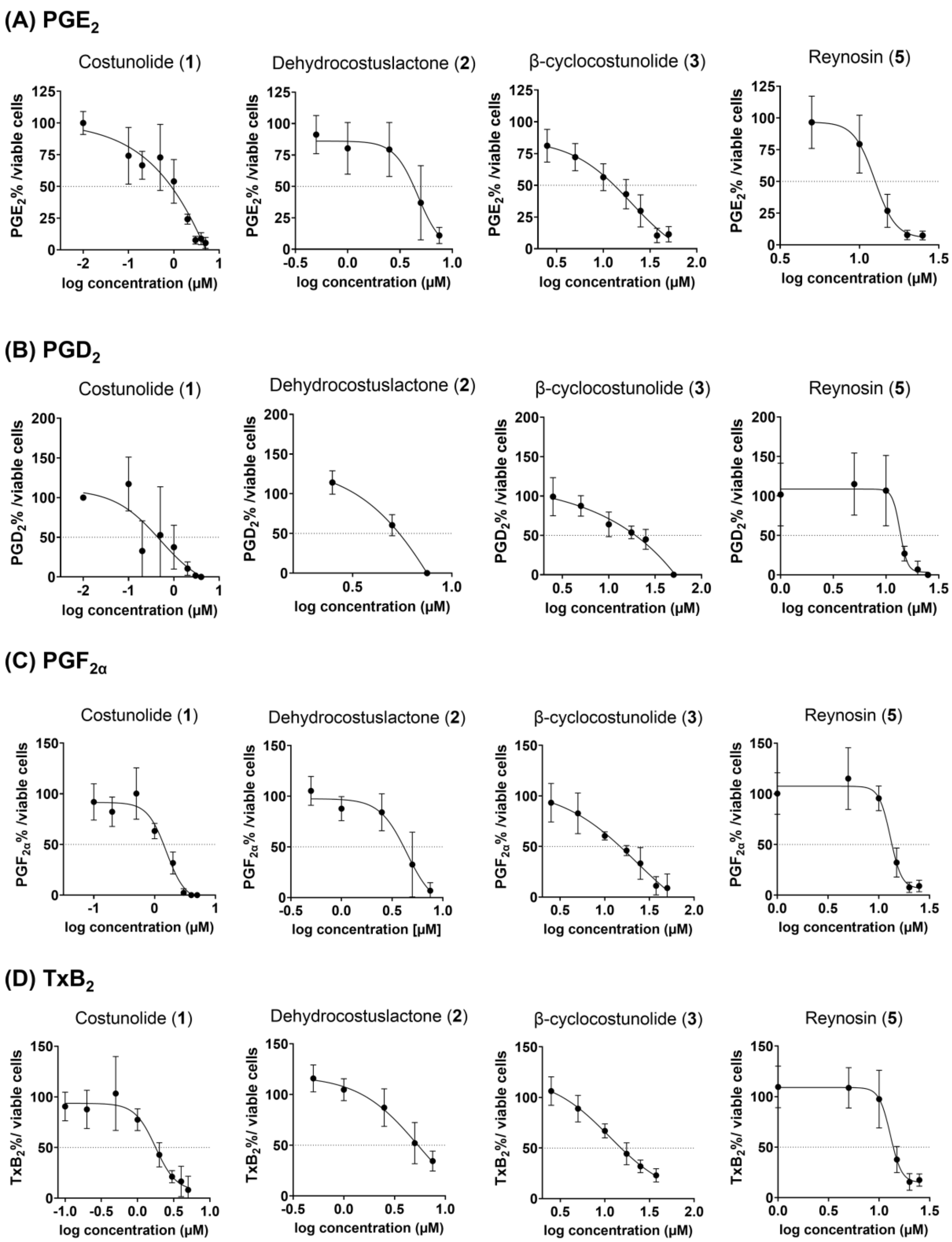

Figure S7: Dose-response curves of isolated active compounds on prostaglandin levels in the SW-NF-κB cell assay: PGE_2 (A), PGD_2 (B), $PGF_{2\alpha}$ (C), and TxB_2 (D).

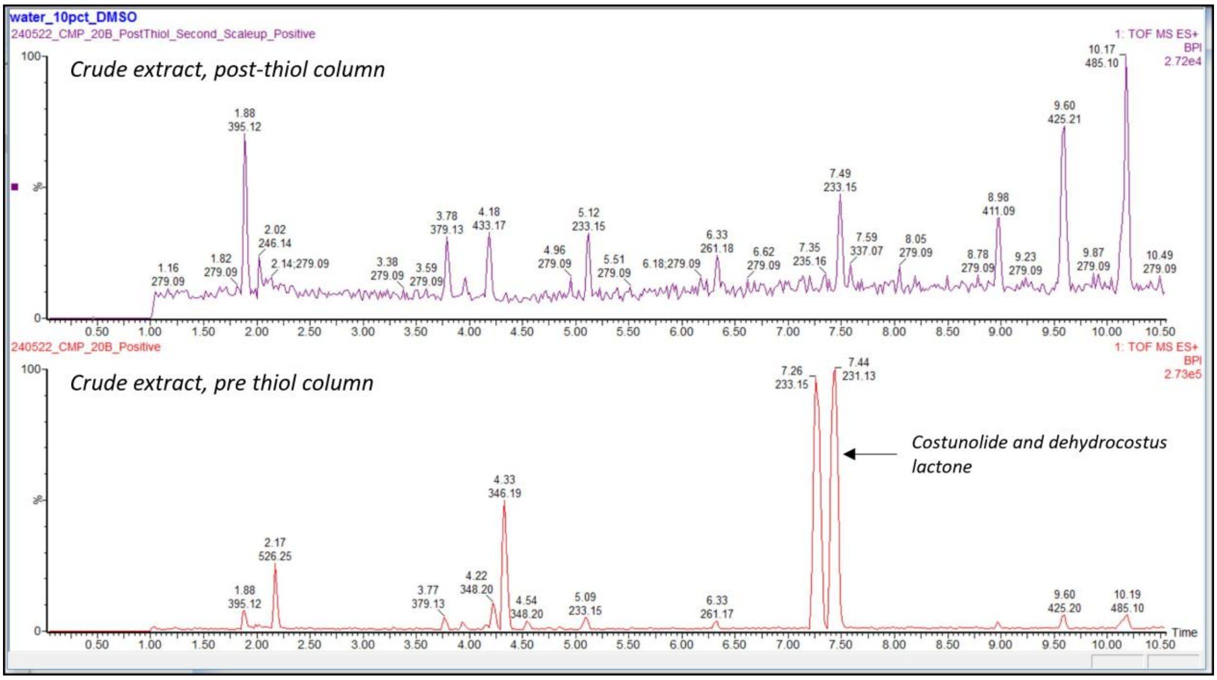

Figure S8: LC-MS comparison of the extract after eluting through silica-bonded thiol (top) with the crude extract (bottom). Peaks associated with the major sesquiterpene lactones costunolide (**1**) and dehydrocostus lactone (**2**) are marked.

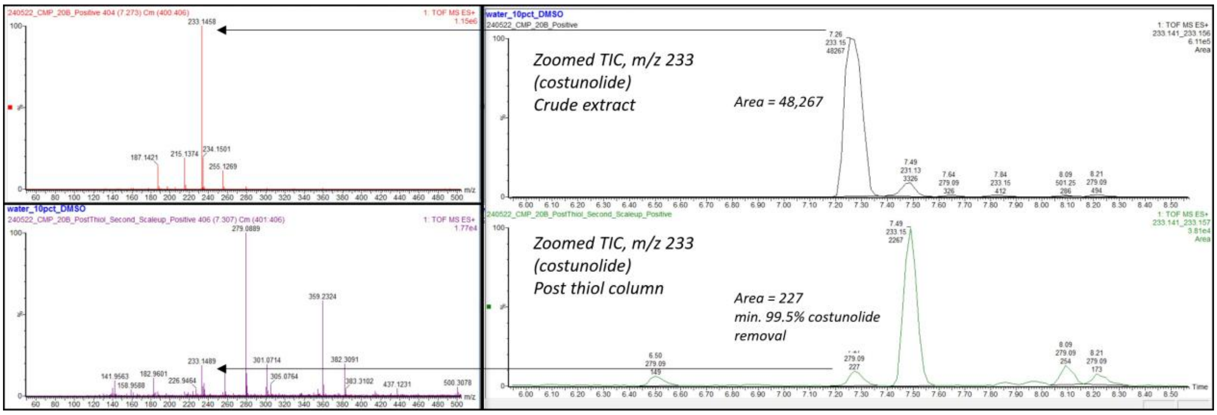

Figure S9: Zoomed LC-MS TIC (m/z 233, costunolide) of the extract after eluting through silica-bonded thiol (bottom) with the crude extract (top). Integrating these shows >99.5% removal of costunolide.

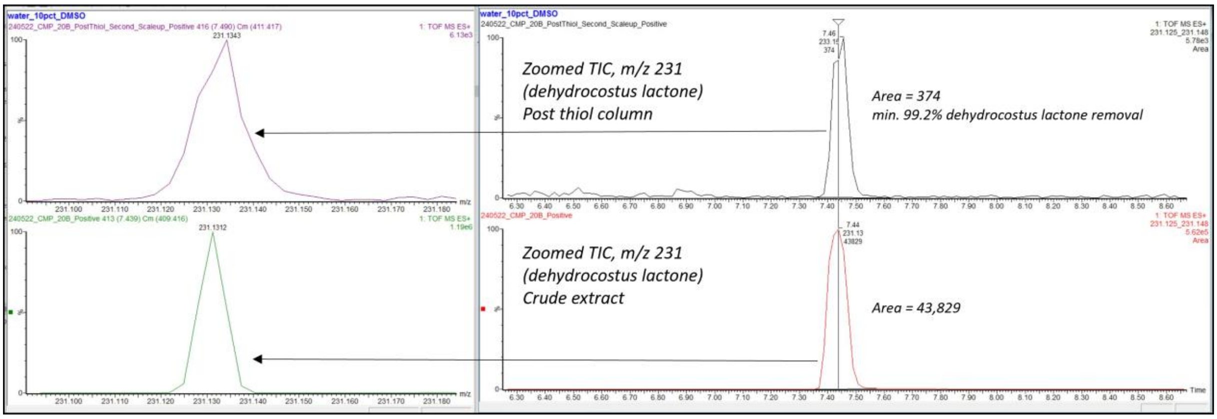

Figure S10: Zoomed LC-MS TIC (m/z 231, dehydrocostus lactone) of the extract after eluting through silica-bonded thiol (top) with the crude extract (bottom). Integrating these shows >99.2% removal of dehydrocostus lactone.

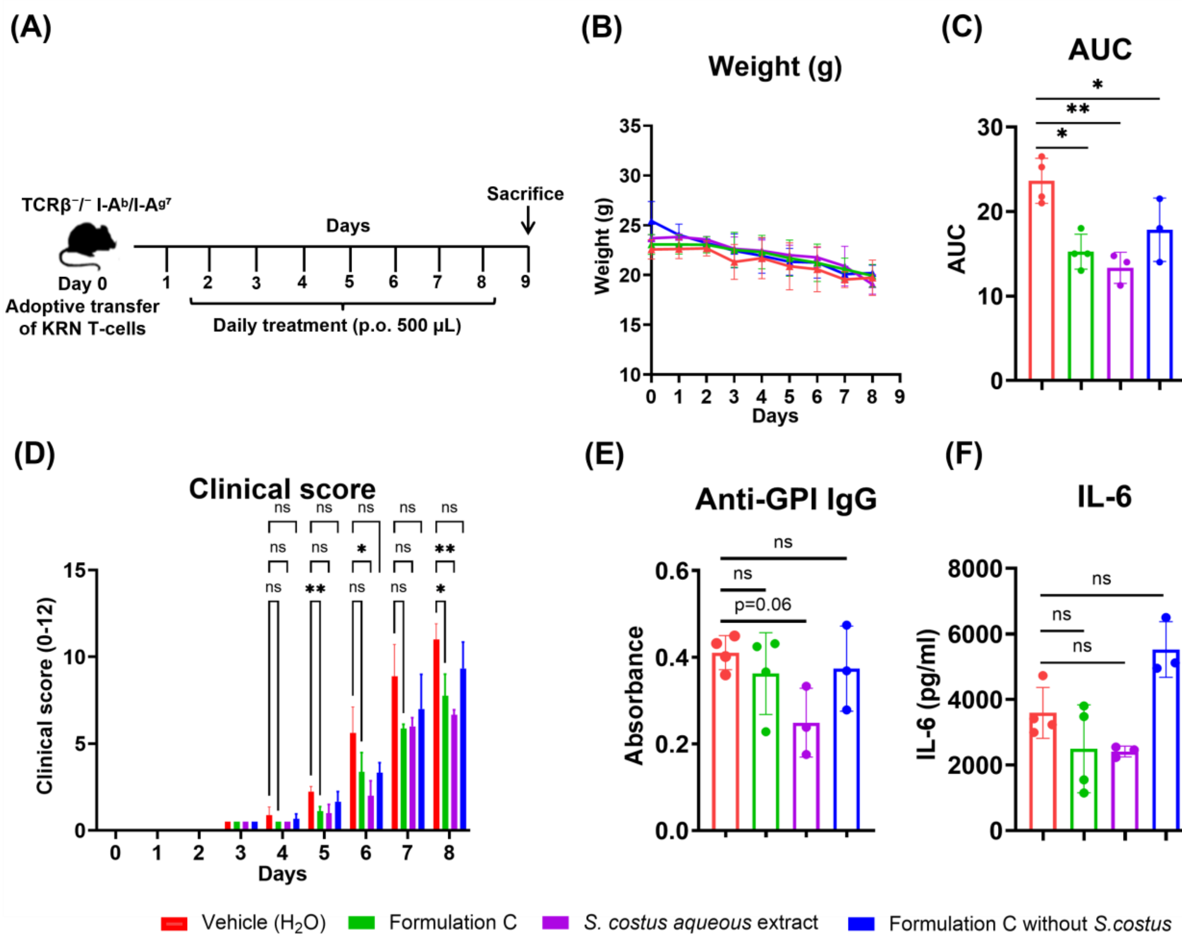

Figure S11: **Formulation C and the aqueous extract of *S. costus* reduce joint swelling in the KRN arthritis model**. Mice were randomly assigned to one of the following groups: Vehicle (n=4), Formulation C (n=4), *S. costus* aqueous extract (n=3), or Formulation C with *S. costus* removed (n=3). (**A**) Induction of arthritis and treatment scheme. (**B**) Body weight. (**C, D**) Arthritis severity was assessed daily by an investigator blinded to the treatment. (**E, F**) At the endpoint, serum was analyzed for anti-GPI IgG antibody (E) and IL-6 levels (F) by ELISA. Data is presented as mean ± SD. * $p<0.05$, ** $p<0.01$, and ns = not significant by two-way ANOVA (**D**) and one-way ANOVA (**C, E, F**).

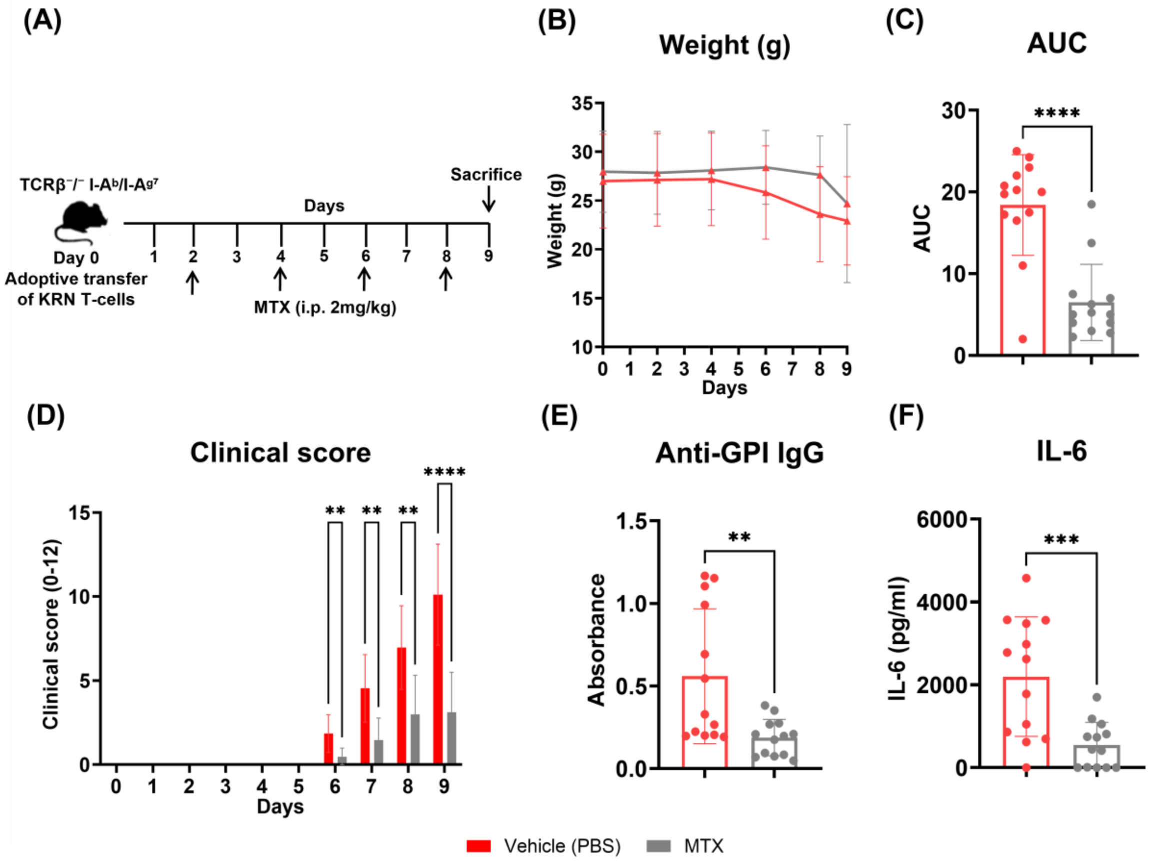

Figure S12: **Methotrexate reduces joint inflammation in the KRN arthritis model.** Mice were randomly assigned to one of the following groups: Vehicle (n=13), or MTX (n=13). (**A**) Induction of arthritis and treatment scheme. (**B**) Body weight. (**C, D**) Arthritis severity was assessed daily by an investigator blinded to the treatment. (**E, F**) At the endpoint, serum was analyzed for anti-GPI IgG antibody (E) and IL-6 levels (F) by ELISA. Data is presented as mean ± SD, pooling two independent experiments. * $p<0.05$, ** $p<0.01$, and ns = not significant by two-way ANOVA (**D**) and one-way ANOVA (**C**), and unpaired t-tests (**E, F**).
